# The Pampa del Indio project: District-wide quasi-elimination of *Triatoma infestans* after a 9-year intervention program in the Argentine Chaco

**DOI:** 10.1371/journal.pntd.0011252

**Published:** 2023-04-24

**Authors:** Ricardo Esteban Gürtler, María Sol Gaspe, Natalia Paula Macchiaverna, Gustavo Fabián Enriquez, Lucía Inés Rodríguez-Planes, María del Pilar Fernández, Yael Mariana Provecho, Marta Victoria Cardinal

**Affiliations:** 1 Universidad de Buenos Aires. Facultad de Ciencias Exactas y Naturales, Laboratorio de Eco-Epidemiología, Buenos Aires, Argentina; 2 CONICET-Universidad de Buenos Aires, Instituto de Ecología, Genética y Evolución de Buenos Aires (IEGEBA), Buenos Aires, Argentina; 3 Instituto de Ciencias Polares, Ambiente y Recursos Naturales, Universidad Nacional de Tierra del Fuego, Ushuaia, Argentina; 4 Paul G. Allen School for Global Animal Health, Washington State University, Pullman, Washington, United States of America; 5 Ministerio de Salud de la Nación, Dirección de Control de Enfermedades Transmitidas por Vectores, Buenos Aires, Argentina; Instituto Oswaldo Cruz, BRAZIL

## Abstract

**Background:**

The elimination of *Triatoma infestans*, the main domestic vector of *Trypanosoma cruzi*, is lagging behind expectations in the Gran Chaco region. We implemented an insecticide-based intervention program and assessed its long-term effects on house infestation and bug abundance in a resource-constrained municipality (Pampa del Indio, northeastern Argentina) inhabited by creole and the Qom indigenous people (2007–2016). Key questions were whether district-wide data integration revealed patterns concealed at lower spatial levels; to what extent preintervention infestation and pyrethroid resistance challenged the effectiveness of insecticide-based control efforts, and how much control effort was needed to meet defined targets.

**Methods:**

Supervised vector control teams i) georeferenced every housing unit at baseline (1,546); ii) evaluated house infestation using timed-manual searches with a dislodging aerosol across four rural areas designated for district-wide scaling up; iii) sprayed with pyrethroid insecticide 92.7% of all houses; iv) periodically monitored infestation and promoted householder-based surveillance, and v) selectively sprayed the infested houses, totaling 1,823 insecticide treatments throughout the program.

**Results:**

Baseline house infestation (mean, 26.8%; range, 14.4–41.4%) and bug abundance plummeted over the first year postintervention (YPI). Timed searches at baseline detected 61.4–88.0% of apparent infestations revealed by any of the methods used. Housing dynamics varied widely among areas and between Qom and creole households. Preintervention triatomine abundance and the cumulative frequency of insecticide treatments were spatially aggregated in three large clusters overlapping with pyrethroid resistance, which ranged from susceptible to high. Persistent foci were suppressed with malathion. Aggregation occurred mainly at house compound or village levels. Preintervention domestic infestation and abundance were much greater in Qom than in creole households, whereas the reverse was recorded in peridomestic habitats. House infestation, rare (1.9–3.7%) over 2–6 YPI, averaged 0.66% (95% confidence interval, 0.28–1.29%) at endpoint.

**Conclusions:**

Upscale integration revealed multiple coupled heterogeneities (spatial, sociodemographic and biological) that reflect large inequalities, hamper control efforts, and provide opportunities for targeted, sustainable disease control. High-coverage, professional insecticide spraying combined with systematic surveillance-and-response were essential ingredients to achieve the quasi-elimination of *T*. *infestans* within 5 YPI and concomitant transmission blockage despite various structural threats and constraints.

## Author summary

The Gran Chaco ecoregion is the main hotspot of Chagas disease. We assessed the district-wide effects of an insecticide-based intervention program on house infestation with the main vector *Triatoma infestans* in rural villages of Pampa del Indio municipality, in the Argentine Chaco. The program included periodic assessments of house infestation and household demographics; blanket house spraying with pyrethroid insecticide at the outset; promotion of householder-based surveillance, and systematic vector surveillance-and-response over a 9-year period. We found that house infestation plummeted over the first year postintervention (YPI); became rare over 2–6 YPI, and fell below 1% at endpoint. House infestation was spatially aggregated in three large clusters that partially overlapped with pyrethroid resistance. Triatomine aggregation occurred mainly at house compound or village levels. Indigenous households were at much greater risk of vector-borne transmission than creole households. We conclude that gradual scaling up of high-coverage, professional insecticide spraying combined with systematic surveillance-and-response operations within an adaptive management framework can achieve the quasi-elimination of *T*. *infestans* and concomitant blockage of vector-borne transmission in resource-constrained, remote rural settings.

## Introduction

The elimination of neglected tropical diseases (NTD) have gained momentum since the 1990s [1]. Major examples of regional elimination programs of vector-borne NTDs are those of onchocerciasis targeting simulid blackflies (vectors of river blindness) in Africa and the Americas [2]; tsetse flies (vectors of sleeping sickness or African Trypanosomiasis) [3], malaria in the Americas, and two species of triatomine bugs (*Triatoma infestans* and *Rhodnius prolixus*) involved in the transmission of *Trypanosoma cruzi*, the etiologic agent of Chagas disease [4,5].

Chagas disease potentially affects 25–30% of the 5–7 million people estimated to be infected with *T*. *cruzi* [6]. Disease prevention has relied mostly on residual spraying with insecticides to suppress domestic infestations with triatomines; screening of blood donors, and etiologic treatment of acute and early chronic infections with benznidazole or nifurtimox [7,8]. The introduction of residual organochlorine insecticides to combat triatomines in the late 1940s and of regional or nation-wide Chagas disease control programs over the next two decades paved the way for subsequent progress. Several intergovernmental initiatives were created over the 1990s to interrupt vector-borne and transfusional transmission of *T*. *cruzi* at regional scales [7]: the Southern Cone (1991); the Andean Pact (1997); Central America (1997), later joined by Mexico, and the Amazon basin initiative (2004). A large number of reports glossed over program trajectories over time [9–15]. A major recent achievement was the elimination of *R*. *prolixus* from Central America, where it had been introduced by 1915 and had no sylvatic foci [5].

The Southern Cone initiative set among its goals for the year 2000 “to eliminate *T*. *infestans* of dwellings and peridomestic ecotopes of endemic and probably endemic areas” except where *T*. *infestans* had sylvatic foci, such as in Bolivia [4]. The regional elimination of *T*. *infestans*, pioneered by the State of Sao Paulo (Brazil) in 1990, was subsequently met by 10 of the 12 states invaded by this species since the early 1900s [16]. The Southern Cone program gradually certified the interruption of vector- and blood-borne transmission of *T*. *cruzi* in Uruguay, Chile, Brazil, Paraguay, and in several districts of Argentina and Bolivia.

Progress was slower in the Gran Chaco ecoregion, a hotspot of NTDs extending over 1.5 million km^2^ across Argentina, Paraguay and Bolivia, and the most affected by Chagas disease in the Americas [17]. Argentina ranked at the top of the affected countries with roughly 1.5 million infected people; 3% of infected blood donors, and roughly 1000–1500 congenital cases occurring every year as of 2010 [18]. This state of affairs can be traced back to past conditions when sporadic insecticide spraying campaigns were rarely followed by systematic vector surveillance-and-response. A mass serosurvey of 1.8 million young men drafted into military service over 1981–1993 showed large, though heterogeneous, declines in the seroprevalence of *T*. *cruzi* and house infestation across provinces over time [19]. The ranking of human infection was led by two provinces in the core of the Gran Chaco region: Chaco and Santiago del Estero. In Chaco province, 36.5% of 18-year-old draftees were seropositive for *T*. *cruzi* in 1980 [20] and decreased to 13.5% in 1993 [19]. More recent serosurveys in rural communities displayed high prevalence rates of human infection with *T*. *cruzi* ranging from 25 to 57%, especially in Qom, Wichi and other indigenous populations [21–27].

The consensus at the outset of the Southern Cone Initiative was that the main threats to the regional elimination of *T*. *infestans* and vector-borne transmission were the “unusual house construction features such as the dense roofs of brushwood and packed earth of some houses in the Chaco region”, and the occurrence of sylvatic foci of *T*. *infestans* (only known in central Bolivia at that time) and of non-target secondary triatomine species with extensive sylvatic foci [4: p. 20]. Some biological features of *T*. *infestans* suggested it was an unlikely candidate to develop pyrethroid resistance. The threat of house reinfestation originating from peridomestic habitats (frequently infested with large populations of *T*. *infestans*) was omitted, and so was the fact that pyrethroids were substantially less effective in outdoor peridomestic structures [28]. In practice, high levels of pyrethroid resistance emerged in northern Argentina and southern Bolivia by the late 1990s [29,30]. Extensive, widespread sylvatic foci of *T*. *infestans* were detected throughout Bolivia [31] and Chile [32], with more discrete occurrences in Argentina and Paraguay [33–35]. Long-term longitudinal studies revealed that no secondary triatomine species (e.g., *Triatoma guasayana* and *Triatoma sordida*) were able to establish bug colonies in domestic habitats after the local suppression of *T*. *infestans* despite being common in the immediate surroundings of rural houses [36–38]. *Triatoma sordida*, a subcomplex of cryptic species including *Triatoma rosai* from northeastern Argentina [39], offered one remarkable exception at one location in Bolivia [40].

By the year 2005, it became clear that the elimination of *T*. *infestans* had met unexpected challenges. The standard methods in practice were apparently less effective in large sections of the Gran Chaco for reasons not completely understood at that time [41]. Obstacles for the sustained control of *T*. *infestans* in this region also have political and socioeconomic roots: structural poverty (especially in rural populations); lack of adequate infrastructure and recurrent socioeconomic crises; disorganized decentralization of health service programs, and the emergence of dengue outbreaks [17], more lately followed by other emerging mosquito-borne viral diseases (Zika and Chikungunya). A common denominator in much of this region has been the lack of sustainability of vector control efforts leading to repeated cycles of house infestation-suppression-invasion and re-establishment of domestic triatomine colonies leading to heterogeneous patterns of vector control status and parasite transmission. Chagas disease was targeted for elimination as a public health problem in the 2020–2030 roadmap [1]. However, “…, research remains critical to address questions pertaining to how to achieve elimination with currently available tools and especially to how to optimize implementation in different epidemiological, sociocultural, and health system settings” [42]. Whether *T*. *infestans* can be suppressed from the Gran Chaco ecoregion and whether such status can be sustained has rarely been addressed; both problems merit research at multiple scales. The neglect reflects in the scarcity of detailed long-term studies of house infestation in areas with pyrethroid resistance [43].

A multi-country research project sponsored by Tropical Disease Research (TDR/WHO) sought to identify the main sources of house reinfestation with major Chagas disease vectors after insecticide spraying across the Gran Chaco and the Brazilian *cerrado* and *caatinga* ecoregions [35,44,45]. Following its implementation in a rural section of Pampa del Indio (Chaco Province), the Argentine arm scaled up the interventions to the municipality-wide level (divided in four operational areas) to test whether intensified, high-quality vector control actions were able to suppress *T*. *infestans* from all rural villages in a sustained fashion and to interrupt the domestic transmission of *T*. *cruzi* infection before launching mass etiologic treatment of *T*. *cruzi*-seropositive children. The program was implemented in the context of persistently infested adjacent municipalities (henceforth, districts) subjected to widely variable levels of pyrethroid-based control and (much later revealed) emerging high resistance to pyrethroids [46]. Supervised evaluations of house infestation and spraying with pyrethroids combined with community-based triatomine surveillance largely reduced house infestation over the first year postintervention (YPI) across the three main areas [47–49]. In general, domestic infestation was closely associated with type and quality of house construction, refuge availability for triatomines and indoor insecticide use, whereas most peridomestic foci occurred in chicken coops and other habitats occupied by chickens. The seroprevalence of human infection with *T*. *cruzi* at baseline ranged from 29.0% to 39.8% and was directly related to infected-bug abundance at house level [26,27]. Several vector-based indices correlated positively with summary measures of host availability and social vulnerability–a composite of substandard housing, overcrowding, educational and income levels strongly linked to Qom households [50]. Vector surveillance and selective insecticide sprays retained house infestation below short-term target levels (5%) over 2–6 YPI across areas 1–3 [45,48,49]. Triatomine abundance was spatially and temporally heterogeneous across multiple rural villages [51]. Several *T*. *infestans* populations showing mainly incipient to moderate pyrethroid resistance caused control failures in two distant areas [47,49]. The outcomes of district-wide serosurveys of human and canine infection with *T*. *cruzi* conducted over 9–10 YPI combined with historical house infestation information supported the apparent interruption of parasite transmission to humans across rural settings of Pampa del Indio [52].

Here, we report the district-wide scaling up of control interventions and its long-term impacts on house infestation and abundance of *T*. *infestans* stratified by operational area and major vector habitats (domestic, peridomestic) over nearly a decade. In doing so, we provide a detailed account of surveillance-and-control efforts (inputs) that reduced house infestation and triatomine abundance (outputs) and led to the apparent interruption of vector-borne transmission to humans (outcome) described above. We extended the time series of vector control actions and house infestation data across 2007–2016 for all four areas (including previously unpublished data for area 4), and integrated this information into a common timeline to assess the spatial distribution of entomological indices of human infection risk and cumulative control effort over time at a district-wide scale. Vector infection with *T*. *cruzi* will be reported separately. Key questions were whether upscale integration of household demographic features, infestation indices and control efforts revealed new patterns that were concealed at more detailed spatial and temporal scales (e.g., spatial aggregation of triatomine abundance and the relationship between household ethnicity and infestation), and to what extent the emerging levels of resistance detected challenged the immediate and long-term effectiveness of pyrethroid spraying.

We also investigated the relationship between house infestation indices and the frequency of insecticide applications over time postintervention. This is a fundamental question rarely addressed in the literature of Chagas disease control [53–56]: how much control effort is needed to meet defined intermediate outputs, such as house infestation prevalence below 5% (to enter the surveillance phase with selective house treatments) or 1%, in the pathway towards the endpoint program outcome (e.g., suppressing vector-borne transmission of human infection). This has been denominated the effort-outcome principle, action-response curve or investment-outcome relationship, among others [57]. The links among inputs, outputs and program outcomes provide relevant information for the sustained elimination of *T*. *infestans* and other major domestic triatomine species targeted for control. This paper may be the first to provide detailed results of decade-long interventions to suppress *T*. *infestans* at a district-wide scale in the Gran Chaco and link these outputs to the apparent interruption of parasite transmission to humans.

## Materials and methods

### Ethics statement

The study protocol was approved by the Dr. Carlos A. Barclay Independent Ethical Committee for Clinical Research, Buenos Aires, Argentina (IRB No. 00001678; Protocol N° TW-01-004, Revision N° 863-32-2011).

### Study area

**Fig 1:**
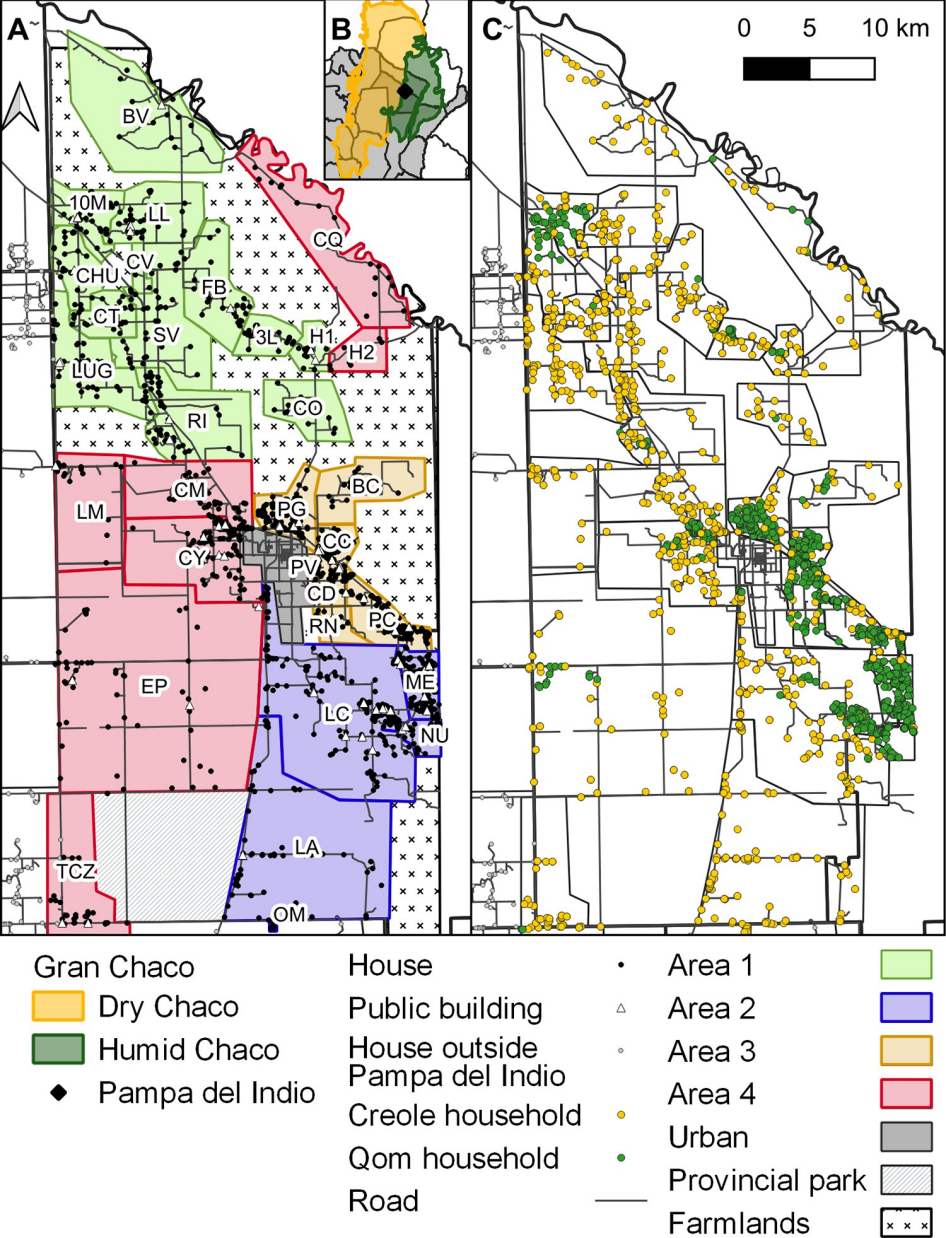
Distribution of villages, housing units and household ethnicity in Pampa del Indio. Operational areas are shown as colored-shaded polygons (A). Inset B shows the location of Pampa del Indio Municipality within Chaco Province and the Gran Chaco ecoregion. Distribution of household ethnicity (C). Maps were based on the data collected within the scope of this study and using base layers from Instituto Geográfico Nacional (Argentina) at: https://www.ign.gob.ar/NuestrasActividades/InformacionGeoespacial/CapasSIG\h. The maps were created in QGIS 2.18.11. Village acronyms: 10M, 10 de Mayo; CT, Campo Los Toros; CO, Colonia Ombú; SV, El Salvaje; CV, Los Ciervos; FB, Fortín Brown; H1, La Herradura; LL, La Loma; BV, Las Bravas; CHU, Las Chuñas; RI, Santa Rita; LUG, Santos Lugares; 3L, Tres Lagunas; LC, Lote Cuatro; CN, Campo Nuevo; ME, Campo Medina; LA, Cancha Larga; OM, Pampa Ombú; BC, La Barrancosa; PG, Pampa Grande; PC, Pampa Chica; CC, Cuarta Legua Catorce; CD, Cuarta Legua Diecisiete; PV, Pueblo Viejo rural; RN, El Rincón; CQ, Campo Cacique; H2, La Herradura 2; CY, Campo Alemany; CM, Colonia Mixta; LM, Las Muñecas; EP, ex-Parque; TCZ, Tacuruzal.

The municipality of Pampa del Indio (San Martín Department, Chaco Province), extending approximately 60 km by 30 km, comprised a urban core (Pampa del Indio and Pueblo Viejo), a fast-growing peri-urban section (Parque Industrial), and 32 dispersed rural settlements with diffuse limits as of 2007 (Fig 1A and 1B). The frequency of houses per area at baseline (2007–2009) varied widely (range, 280–437). House density tended to decrease with increasing distance from the urban center, with more compact rural settlements (both creole and Qom) occurring in its proximity and route-related gradients. For vector control purposes, we partially included adjacent houses from small rural villages that extended over adjacent jurisdictions to the west and south (i.e., Tacuruzal, shared with Quitilipi and Maipú Departments, and Santa Carmen and 10 de Mayo, part of General Güemes Department) and Pampa Ombú (in 25 de Mayo Department). Two municipality borders lacked any adjacent population settlement: the Bermejo River to the north, and extensive farmlands to the east.

Pampa del Indio ranked among the top 50 municipalities with higher levels of social deprivation in Argentina. The 2010 census enumerated 15,287 people in 3,862 housing units, and a large annual population growth rate (4.9%) over the previous decade. The district is inhabited by two main groups: Qom, a nomadic or semi-nomadic hunter-gatherer indigenous people that settled down by the early 1900s, and creoles of European descent that arrived at that time. At baseline, most houses had mud walls and thatched or corrugated tarred cardboard roofing; they were more often infested than those with brick-and-cement walls and tin roofs [49,58,59]. Qom households were larger and lived in small-sized, recently-built, precarious houses with fewer peridomestic structures and livestock than creoles. Rural houses usually lacked any service, except those close to the local town, which had electricity and water supply. These conditions tended to progress toward the endpoint [48] though at a different pace among areas.

Pampa del Indio lies exactly on the transition between the dry (west) and humid (eastern) Argentine Chaco [60]. Annual mean temperature is 23°C (mean annual minimum and maximum temperatures, 17 and 29°C, respectively). Annual rainfall averages 954 mm concentrated from October to April, with wide inter-annual fluctuations including extraordinary floods and draughts. The relief is flat and the landscape is a mosaic including gallery forests along water courses, small forest fragments, preserved native dry forest (at the Provincial Park of Pampa del Indio, 8,366 ha), crop fields (mainly cotton, corn, pumpkin and soybean) and grasslands. Rural residents live mostly on a subsistence economy based on agriculture and raising livestock (mainly goats and more rarely cattle or sheep), and largely depend on governmental welfare programs.

The Chagas disease control program of Chaco appointed Pampa del Indio as the study site for a long-term intervention program in July 2007 because of its high levels of house infestation with *T*. *infestans* and lack of insecticide spraying campaigns since 1996–1999. Local health-care personnel selectively sprayed with insecticide a few small sections over 2000–2008 [49,59,61]. A few external aid organizations occasionally assisted with insecticide donations to selected rural villages. According to Chaco vector control records over 2003–2012, all districts adjacent to Pampa del Indio displayed high house infestation rates before and during our study and were subjected to sporadic insecticide spraying campaigns.

### Definitions

We use the following language conventions [51]. We use "areas" to refer collectively to the four operational areas (1–4) in which we divided rural Pampa del Indio (Fig 1A). A “household” is defined as all the people who occupy a housing unit including related and nonrelated family members; in our specific context one household equaled one housing unit. A “house compound” consists of a domicile or domestic habitat (i.e., a separate structure used as human sleeping quarters) and a peridomestic habitat comprising a patio and other near-by buildings for human and animal use (e.g., kitchens, storerooms, chicken coops). House compounds sometimes had more than one separate domicile used as sleeping quarters by related family (i.e., extended family). We use "habitat" (or ecotope) for a category of individual places that were surveyed for triatomines (e.g., chicken coop, goat corral). We use "site" for a particular exemplar of a habitat, such as a particular chicken coop. Throughout, “intervention” refers to the initial area-wide insecticide spraying (attack phase); postintervention surveys were conducted at some defined time after initial spraying (i.e., months or years postintervention: MPI and YPI, respectively). The term “infested” applies to any site, house, or village in which live *T*. *infestans* (except eggs) were collected. “Creoles” include white people of European descent and in Argentina is currently used for people whose ancestors were already present during the colonial period regardless of their ethnicity barring dark-skinned people of African descent.

### Study design

The intervention program was designed to assess the effects of conventional area-wide spraying with pyrethroid insecticide (attack phase), followed by systematic vector surveillance-and-response, on house infestation and abundance of *T*. *infestans* in the context of persistently infested neighboring districts. This standard approach before the 1990s differed in two important respects from the typical procedures applied under the aegis of the Southern Cone Initiative: first, rather than using two rounds of house spraying with pyrethroids aiming at full coverage, the insecticide was applied once to all housing units, leading to monitoring of infestations and selective insecticide sprays within 4–14 MPI; and second, triatomine surveys and spray operations were conducted under the supervision of a research team member. The underlying premises at program onset were that in the absence of pyrethroid resistance, supervised insecticide applications would suppress the majority of existing infestations at a lower cost than using two full-coverage insecticide rounds, and that any residual foci would be detected and treated on the next rounds of surveillance-and-response, causing a rapid initial impact and then a gradual decline toward local vector suppression. Given the limited detectability of timed-manual searches to detect low-density house infestations, household-based triatomine surveillance combined with repeated manual searches would reveal any new foci in due time.

The intervention program included a district-wide exploratory (pilot) survey for geographical reconnaissance (in 2007); an area-wide survey of house infestation with triatomines and household sociodemographic and environmental characteristics at baseline; house spraying of pyrethroid insecticide aiming at full coverage; scaling up of program activities until covering the four areas over 2007–2010, and periodic triatomine surveillance including selective insecticide spraying of detected foci (over 2008–2016). These activities were complemented with community meetings and workshops conducted at local primary health-care posts, churches or schools to mobilize householders for triatomine surveillance, subsequent serosurveys and etiological treatment rounds as described [26,27,62]. We applied the same intervention protocol in the four areas.

### Vector surveys

#### Geographical reconnaissance

As detailed village-level sketch maps were virtually unavailable except at a few rural health-care posts, we conducted a district-wide exploratory survey to collect preliminary data on village location, roads and accessibility, house infestation and sociodemographic characteristics; identify the triatomine species infesting houses, and assess their infection status with *T*. *cruzi* in September 2007. The goal was to select a well-defined rural section with approximately 300 houses for detailed interventions; the nearest houses of external villages were at >1 km. The exploratory survey was indispensable for geographical reconnaissance, identification of priority areas and scaling up operations.

Two teams, each including three technicians accompanied by a local referent, assessed house infestation with triatomines in a convenience sample of the main rural villages across Pampa del Indio. In total, 111 houses from 21 villages were surveyed for triatomines using timed-manual searches assisted with 0.2% tetramethrin (Espacial, Buenos Aires, Argentina); the count excludes houses that were subsequently dismantled or could not be relocated and houses from adjacent districts. Each domicile and peridomestic structure was inspected during approximately 15 min by one skilled bug collector. All bugs collected in a site were stored separately in a labelled, self-sealing plastic bag; identified taxonomically and counted according to species, stage or sex at the field laboratory, and examined individually for *T*. *cruzi* infection as described [58]. Based on the observed infestation in the exploratory survey, the rural sections of Pampa del Indio were divided into four areas (1–4) for scaling up assessment and control actions, with area 1 selected for detailed research.

#### Baseline survey

At the initial visit to a household while accompanied by local health-care or vector control personnel, we explained to householders the goals of the research and the planned interventions using a simple language and requested their permission to access the premises. Each housing unit and public building was identified with an aluminum numbered plate at the main entrance, and its location georeferenced with a GPS receiver (Trimble GeoXM or Garmin Legend). We drew a sketch map of the spatial distribution of all structures within the house compound including approximate distance between sites for subsequent identification across the follow-up. Each household was further identified by the full name of its head.

We used three methods to assess house infestation with triatomine bugs: i) timed-manual searches assisted with an aerosol to dislodge the insects (0.2% tetramethrin); ii) householder collection of any triatomine they sighted into the provided plastic bags, and iii) insecticide spray-related triatomine collections, including bugs spotted during or after house spraying. In some infested houses, the stipulated search time was extended to increase sample size for blood-meal identification and wing geometric morphometry; these data and householder bug collections or through other methods were not used to compute triatomine abundance. Search teams included one supervisor and two or three skilled bug collectors. Each domestic or peridomestic site was inspected for triatomines by one person during approximately 15 min. Closed houses were usually re-visited once or twice at times recommended by neighbors. Public buildings (nonresidential) were inspected for triatomines at baseline and occasionally thereafter if its infestation status was in doubt; as hardly any of them was ever found to be infested they were excluded from further inspection except when a fraction of it was used as living quarters of one or more individuals. Vacant houses to which we were allowed access were inspected for triatomines. All bugs were processed as before.

House infestation with *T*. *infestans* was determined by the finding of at least one live bug (except eggs) by timed-manual searches at three defined habitat levels (house compound, domicile(s), and all peridomestic sites pooled together) or by any of the three methods used. Triatomine relative abundance was calculated as the total number of live triatomines caught by timed-manual searches per unit effort across all inspected habitats (in practice, this averages approximately one person-hour per house over four inspected sites per house compound); triatomine abundance was estimated at the three habitat levels described above. All metrics of infestation and triatomine abundance were computed for all inspected units and for occupied or vacant houses as specified in each case.

For the sociodemographic survey, we canvassed an adult household member fluent in Spanish (or were assisted by a Qom local referent) and registered the full name of each head of household; use of insecticides indoors and date of the latest insecticide spraying of house premises conducted by vector control personnel using manual compression sprayers; other demographic data, and household ethnicity [48–50]. The ethnic group of the household was assigned on the basis of whether they spoke Qom language; participated in traditional Qom organizations, and took into account the tenants’ physical features and cultural practices. The few households (< 5%) formed by at least one person self-identified as Qom and at least one person self-identified as creole were classified as Qom based on self-identification and cultural practices. Ad-hoc consultation with local health-care agents, community leaders and other referents were made to fill in the missing data for household ethnicity.

The environmental survey recorded the materials used in roof and walls in each domicile, presence and type of wall plaster, condition of wall surface, type of floor, number of sleeping quarters, and the availability of refuges for triatomines as described [58]. We also registered the physical characteristics and materials used in each peridomestic structure, its main function and resident host species numbers. The main features of areas 1–3 at baseline were detailed elsewhere [49,58,59]. The average number of human residents ranged from 3.9 to 6.2. Qom households comprised from 16.0% to 89.6% of all listed housing units in each area.

#### Area-wide insecticide spraying

Vector control personnel sprayed the structures of every house compound with suspension concentrate (SC) deltamethrin (at 25 mg/m^2^, K-Othrin, Bayer) or beta-cypermethrin (at 50 mg/m^2^ Sipertrin, Chemotecnica) using standard procedures [63] and backpack manual compression sprayers (Guarany, Brazil, and Hudson, Illinois) immediately after each baseline survey. Treatment criteria, insecticides and doses applied over time are described in S1 Table. Houses whose residents were absent were usually re-visited up to three times for insecticide treatment. Any triatomine encountered while removing furniture and household goods during or immediately after spraying operations by research team members or householders was collected and processed as before (i.e., insecticide spray-related bug collections).

#### Vector surveillance-and-response

The surveillance phase started after area-wide insecticide spraying (>0 MPI), and comprised periodic timed-manual searches (conducted as above) and householder triatomine collections or reports. Householders were also shown dry specimens of *T*. *infestans*, *T*. *sordida* and other Reduviidae (to make sure reports were not misidentifying other true bugs as Triatominae) at every survey when being requested to report the presence or absence of triatomines in their dwellings.

The periodicity of timed searches for triatomines was flexible as part of an adaptive management strategy (S1 Table). Area 1 was subjected to house inspections every 4–6 months for a detailed investigation of the temporal and spatial dynamics of house reinfestation, and because the initial assessments after area-wide insecticide spraying yielded a greater-than-expected infestation rate exceeding the target levels (<5%) over 4–12 MPI. Postintervention house infestation rates in areas 2 and 3 were substantially lower than in area 1 and remained below the target level; therefore, vector surveys were more spaced. Monitoring was deferred and less frequent in area 4, which had a lower prevalence of house infestation before intervention, more scattered houses, and more difficult access. The exact timing and coverage of field operations took into account the recent status of house infestation in the frame of logistic and resource constraints. A district-wide survey aiming at full coverage was conducted over April–May 2016 to assess house infestation right after the late-summer peak in triatomine abundance.

We classified the occasion-specific status of each housing unit at each survey as follows: i) occupied: when the unit is the usual place of residence of an individual or group of individuals (i.e. inhabited), regardless of whether they were temporarily absent; ii) vacant: when no individual is living in it, often corroborated by neighbors (i.e. uninhabited), regardless of whether it contained furniture or not; iii) demolished: no longer existing at its prior location (an absorbing state), and iv) new housing unit: not previously recorded at its georeferenced current location.

During the surveillance phase, all houses in which *T*. *infestans* bugs were caught by timed searches were targeted for selective re-treatment with pyrethroid insecticide of the complete house compound as in the standard intervention protocol. Infested houses that failed to be sprayed with insecticide upon finishing a survey round were re-inspected by timed searches on the subsequent survey and sprayed if *T*. *infestans* was caught on this occasion. When householders returned *T*. *infestans* bugs, the house was thoroughly searched for triatomines and sprayed with insecticides if the infestation was corroborated by timed searches. When timed searches detected *T*. *sordida* in domestic areas, the entire house compound was sometimes sprayed with pyrethroid insecticide depending on circumstantial considerations and householders’ concerns.

In area 1, the early detection of control failures forced us to modify the treatment protocol. All infested sites detected at 4 or 8 MPI and other adjacent sites were selectively re-sprayed with SC deltamethrin at 8 MPI and those detected at 12 MPI were selectively re-sprayed with SC beta-cypermethrin [45,47]. Persistent peridomestic infestations at 8 MPI were randomly assigned to a standard (50 mg/m^2^) or double dose (100 mg/m^2^) spray with SC beta-cypermethrin to assess whether treatment effectiveness outdoors improved whereas domiciles were sprayed with a standard dose [64]. Double-dose insecticide treatments were applied at 17 MPI. All sites within infested house compounds were sprayed from 17 MPI onward for enhanced effectiveness. The few house compounds that were persistently infested with *T*. *infestans* at that time (including a few adjacent houses), jointly with emerging evidence of substantial pyrethroid resistance [45], supported the application of a standard dose of malathion (Onix, Cheminova, Denmark, at 1 g/m^2^) in 15 houses of area 1 over 22–34 MPI and in 2 houses from area 2 at 21 MPI.

#### Pyrethroid resistance

The offspring of 76 *T*. *infestans* populations collected at identified houses or pools of houses across the follow-up period were tested for pyrethroid resistance at the Center for Research on Plagues and Insecticides (CIPEIN/CONICET, Buenos Aires, Argentina) using standardized discriminating dose assays [65] with additional details [48]. First-instar nymphs were individually treated with a 0.2 μl acetone solution of technical-grade deltamethrin (99.0%, Ehrestorfer, Augsburg, Germany) containing a discriminating dose (0.01 mg/ml, equivalent to 0.2 ng/insect) to assess whether each population was resistant or susceptible to deltamethrin. Nearly all assays included three replicates with 10 first instars per population. Mortality was evaluated 24 h after deltamethrin application. Laboratory-reared colonies of *T*. *infestans* used as a negative (pyrethroid-susceptible) and positive control (a pyrethroid-resistant strain from Salta, Argentina) always showed 100% and 0% mortality, respectively. A triatomine population was considered resistant to deltamethrin if mortality was less than 91% in two out of three assays and susceptible otherwise. Pyrethroid resistance level was scored on the basis of bug mortality rates as susceptible (>90%), incipient (76–90%), moderate (45–75%), and high (less than 45%).

### Data analysis

Data management and statistical analyses were conducted using Stata 15.1 [66]. Throughout log is log_10_. S2 Table gives the raw data for each housing unit across the follow-up period, including 19,340 records. As the gradual scaling-up determined that field operations were asynchronic among areas, we used the baseline assessment of house infestation followed by area-wide insecticide spraying as time zero of the intervention program (0 MPI or 0 YPI). The district-wide maps of control actions and outcomes (spraying, infestation) piece together all areas under a common timeline. Maps show maximum triatomine abundance for each occupied or vacant house inspected across occasions over a given time interval.

The exploratory and baseline surveys produced infestation data for 102 houses with a reliable house identification. Houses with missing data for baseline infestation were assigned the bug count assessed at the exploratory survey if available. House infestation metrics additionally included public buildings at baseline and whenever they were used as living quarters. Borderline houses from districts adjacent to Pampa del Indio (eventually inspected and treated) were excluded from current analyses. Therefore, sample sizes for specific areas and periods differ slightly from those reported before and were restricted to occupied or vacant houses unless otherwise noted.

“Detectability or detection probability is the chance of confirming the occurrence of an animal within some defined space and time period” [67,p. 39]. We estimated the apparent detectability of timed-manual searches at house-compound level as the ratio between the number of occupied or vacant houses in which *T*. *infestans* was caught at baseline (*x*) and the number of such houses observed infested using any method at baseline (*y*) (i.e., detectability = *x* / *y*). Alternatively, to allow for previously undetected infestations that eventually emerged subsequently, we added the number of houses in which *T*. *infestans* was caught by any method up to 2 YPI among houses that had been negative by any method (*z*) to *y* (i.e., detectability = *x* /(*y* + *z*). Timed searches for live *T*. *infestans* (barring eggs) can be assumed to have perfect specificity, and hence true prevalence equals apparent prevalence divided by detectability [68].

The association between house-level infestation with *T*. *infestans* and habitat (domestic vs peridomestic), baseline (vs surveillance) infestation status, and household ethnicity (Qom vs creole) stratified by operational area was tested using Cochrane-Mantel-Haenszel (CMH) χ^2^ tests, including χ^2^ tests to assess whether the effect was homogeneous across areas. This analysis included occupied or vacant houses in two separate periods (baseline and the advanced surveillance phase, >14 MPI); the latter period reflected both the combined impacts of the attack phase and selective insecticide treatments during the initial surveys after the attack phase. We used zero-inflated negative binomial regression with robust standard errors to test for household ethnicity effects on triatomine relative abundance (the response variable) at the three habitat levels described above; effect sizes are labeled in Stata output as ‘incidence-rate ratios’, IRR. All two-way interaction terms between household ethnicity and area were non-significant except once (weakly significant) and therefore were excluded from the reported results. The association between pyrethroid resistance levels and area or surveillance-phase period was tested by Fisher’s exact test. The relationship between cumulative spray effort at time *t*-1 and the mean prevalence of house infestation (or bug abundance) at time *t* over MPI was described by ordinary linear regression.

Global spatial analyses used the L(r) function implemented in spatstat package [69]. Random labeling of period-specific maximum triatomine catch, household ethnicity and frequency of insecticide treatments over the surveillance phase was used to test the null hypothesis of random occurrence of marks among the fixed spatial distribution of all houses. The 95% confidence envelope and L(r) values were obtained from the mean of 25 replicates of 999 Monte Carlo simulations. The radial distances selected were 400, 1,000, 2,000 and 5,000 m, approximately reflecting effects within house-compound level, within and between villages (1,000–2,000 m), and at area level, respectively. The time periods considered were 0, 1–3 and >4 YPI. This classification included complete surveys for all areas within the municipality. Heat maps (i.e., density maps) were used to visualize the spatial aggregation of house-level triatomine abundance. The analysis was implemented in QGIS using a kernel density estimation algorithm with radial distances of 400, 1,000, 2,000 and 5,000 m; for illustration purposes we show the 2,000 m outcomes only.

## Results

### Housing dynamics

We registered and georeferenced 2,329 buildings in 32 rural villages throughout the follow-up period, including 1,443 occupied and 103 vacant housing units and 90 public buildings at baseline (range across areas, 269–437), and virtually the same frequency of occupied houses (1,426) and public buildings (88) at the endpoint (S1 Table). However, housing turnover was intense: 693 new housing units were built and 482 were demolished throughout the follow-up (S1 Fig). End-to-end house occupancy (i.e. the frequency of occupied houses at endpoint relative to those occupied at baseline) decreased by 8.5% (300/328) in area 1, by 7.1% (250/269) in area 4 and by 6.4% (409/437) in area 2, while it increased by 14.2% (467/409) in area 3. Qom households were heavily aggregated in areas 2 and 3 and in a few villages elsewhere within the district (Fig 1C).

The frequency of ever leaving a house vacant over the follow-up period (among housing units registered at baseline, excluding public buildings) varied highly significantly among areas, doubling from 17.9% in area 3 to 39.3% in area 1 (χ^2^ = 79.1, *df* = 3, *P* < 0.001). The relative odds of ever leaving a house vacant was significantly lower among Qom rather than creole households (OR = 0.55, 95% confidence interval, CI = 0.40–0.74, CMH χ^2^ = 16.1, *df* = 1, *P* < 0.001), with no evidence of heterogeneity among areas (homogeneity χ^2^ = 4.1, *df* = 3, *P* = 0.25). The fraction of houses that were demolished over the follow-up also differed significantly among areas, increasing from 12.2% in area 4 and 16.9% in area 1 to 22.3% and 26.8% in areas 2 and 3, respectively (χ^2^ = 39.1, *df* = 3, *P* < 0.001). The relative odds of demolishing the house was 2.26 times greater among Qom households across areas (95% CI = 1.59–3.20, CMH χ^2^ = 22.0, *df* = 1, *P* < 0.001; homogeneity χ^2^ = 3.97, *df* = 3, *P* = 0.26).

### Baseline house infestation

The exploratory survey revealed a high prevalence of house infestation with *T*. *infestans* (51.4%, 57/111) across the district and larger infestation in area 1. The time gap between exploratory and baseline surveys offered the opportunity to assess the performance of timed-manual searches of *T*. *infestans* in the absence of insecticide treatment. The inter-survey periods ranged from two months (area 1), 11–13 months (areas 2 and 3) to 26 months (area 4). Among the initially infested house compounds, 80.0% (44 of 55) were again observed positive (infested); one of the 11 initially infested and later negative houses was positive for *T*. *infestans* by other methods. Conversely, 5 (10.6%) of the 47 initially negative houses were subsequently observed infested at baseline. Positive-to-negative transitions included low-density infestations (3.4 bugs per unit effort±0.8 SEM, range 1–10), and so did negative-to-positive transitions except once (7.2±5.7, range, 1–30).

Table 1 shows the apparent detectability of timed-manual searches using a dislodging aerosol in each area over two observation windows (baseline and over the early surveillance period). In area 1 at baseline, 132 (73.7%) of 179 occupied or vacant houses observed positive for *T*. *infestans* by any of the three methods had an infestation revealed by timed searches, whereas none of the 131 negative houses by any method were positive by timed searches. Subsequent inspections for triatomines over 4–12 MPI revealed 64 (all persistent) and 13 (newly observed) infested houses, respectively. Thus, apparent detectability estimates for the two observation windows were 73.7% and 61.4%, respectively. Further extension of the observation period up to 2 YPI revealed no new infestation in always-negative houses. In other areas where the surveys were more spaced, apparent detectability estimates for the two observation windows were 68.4% and 61.9% (area 2); 86.3% and 84.9% in area 3, and 88.0% and 74.6% in area 4, respectively (Table 1).

**Table 1:** Detection of house infestation with *T*. *infestans* by timed-manual searches at baseline and relative detectability by operational area of Pampa del Indio. Includes occupied or vacant houses sprayed with pyrethroid insecticide. Baseline house infestation determined by any method (i.e., timed-manual searches, during insecticide spraying and householder bug collections).

Area	Infested houses by any method at baseline	No. of inspected houses at baseline	No. of houses infested		Apparent detectability	
			by timed searches at baseline	by any method subsequently^a^		
					at baseline	subsequently^a^
1	Yes	179	132	64	73.7	61.4
	No	131	0	13		
2	Yes	38	26	5	68.4	61.9
	No	139	0	4		
3	Yes	124	107	1	86.3	84.9
	No	270	0	2		
4	Yes	50	44	9	88.0	74.6
	No	182	0	9		

^a^ Over 4–12 MPI in area 1 (3 surveys); 14–21 MPI in area 2 (2 surveys); 10–18 MPI in area 3 (2 surveys), and 12–40 MPI in area 4 (2 surveys)

### Control interventions

The program implemented 1,823 insecticide sprays across the 9-year period; the mean ratio of insecticide sprays applied throughout to the frequency of occupied or vacant houses registered at baseline was 1.18 (1823/1546). House spray coverage during the attack phase (0 MPI) across areas comprised 1,355 occupied houses, 74 vacant houses and 70 public buildings, averaging 92.7% of all registered housing units (S1 Table). Spray coverage ranged from 82.3% (area 4) to 98.0% (area 1). Of 84 unsprayed, occupied houses across the district at baseline, 57 were subsequently permanently vacant or repeatedly closed; 24 were *T*. *infestans*-negative and 3 were positive by timed searches; the only positive house that remained unsprayed was unoccupied over 18–63 MPI.

The frequency distribution of house-level insecticide applications over time was strongly aggregated, with most treatments concentrated in the attack phase across areas (S2 Fig). The surveillance phase (>0 MPI) implemented 323 house treatments, ranging from 37 in area 4 to 172 in area 1. These included 24 houses positive for *T*. *sordida* (several of them with indoor catches of adult bugs) and negative for *T*. *infestans* by timed searches. Only 4 houses ever positive for *T*. *infestans* by timed searches were not sprayed with insecticide either immediately or within the next two rounds; all were subsequently observed negative. During the advanced surveillance phase (>14 MPI), householders collected *T*. *infestans* (mainly 1 or 2 adult insects, apparently in-migrants) on 19 occasions in houses that were concurrently negative by timed searches and were not treated with insecticide; as only two of these houses were subsequently found infested, the rest were considered unsuccessful invasion events. On average across the 9-year period, a treated house compound required 3.4 (±SEM, 0.06) monodose units of pyrethroid insecticide. The mean number of monodose units required for each house treatment increased three-fold from 2.8 (±0.1) in area 4, 3.3 (±0.1) in areas 2 and 3, to 7.6 (±0.6) units in area 1.

### Effects on house infestation and triatomine abundance

**Fig 2:**
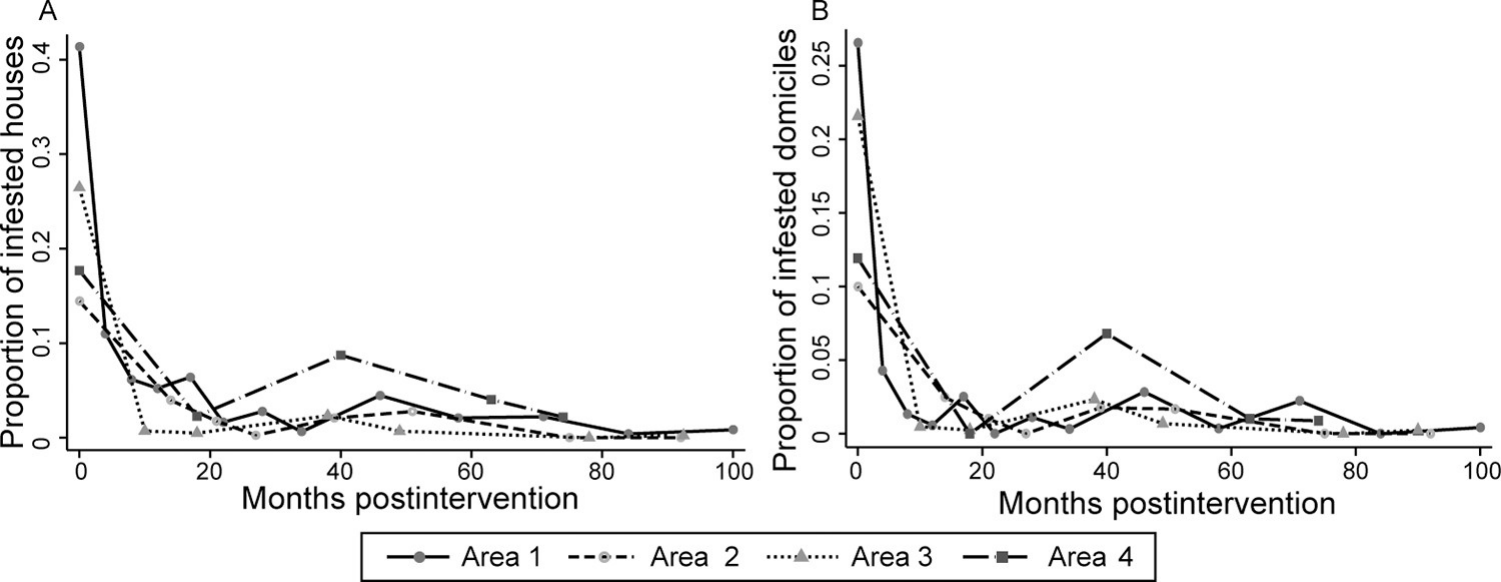
**Prevalence of house infestation with *Triatoma infestans* at house compound-level (A) and in domiciles (B) according to months postintervention and operational area of Pampa del Indio, 2007–2016.** House infestation was determined by timed-manual searches. Includes all housing units inspected for infestation. Maps used base layers from Instituto Geográfico Nacional (Argentina) at: https://www.ign.gob.ar/NuestrasActividades/InformacionGeoespacial/CapasSIG. The maps were created in QGIS 2.18.11. based on the data collected within the scope of this study.

The attack phase caused a steep drop in baseline house infestation (mean, 26.8%; range across areas, 14.4–41.4%) though at a differing pace among them (Fig 2A). In total, 10,410 timed searches for triatomines were conducted over nearly a decade (S1 Table). On 185 occasions householders (from 121 occupied houses) refused to have their premises inspected for triatomines. Householder rejection rate tended to increase slightly from 0.6% (baseline) to 0.2–1.3% over 1–3 YPI, and then fluctuated upwards between 2.1% and 3.4% over 4–7 YPI as infestation became rare. House-compound infestation was rare (1.9–3.7%) over 2–6 YPI and further dropped to 0.66% (8/1,215; exact 95% CI, 0.28–1.29%) at endpoint (2016). The endpoint (apparent) infestation rate was 0.33% (exact 95% CI, 0.09–0.84%) both in domestic and peridomestic habitats. Using a conservative estimate of apparent detectability (0.5) for illustrative purposes, the true prevalence of domestic (or peridomestic) infestation would be 0.66% and the upper limit of the confidence interval would be <1.7%.

In area 1, baseline house infestation (41.4%) determined by timed searches fell gradually from 11.0% to 5.2% over 4–12 MPI, displaying greater-than-expected infestation levels despite selective re-treatments with pyrethroids (Fig 2A). These vector control failures suggested the presence of moderate pyrethroid resistance, further corroborated by discriminant dose assays and field experiments [47]. Infestation dropped below the 5% target at 22 MPI (1.6%), and the remaining highly persistent foci were finally suppressed following one or two treatments with malathion in 15 houses over 22–34 MPI. House infestation then progressively decreased from 4.5% at 46 MPI until reaching 0.8% at 100 MPI.

Areas 2–4 displayed strikingly different patterns from those recorded in area 1 and required fewer selective treatments: house infestation dropped from 14.4% (area 2) and 26.5% (area 3) at baseline to below target levels by 10–14 MPI and remained marginal (<2.8%) until reaching nearly 0% from 75 MPI onwards (Fig 2A). Selective treatments with malathion were conducted in two area-2 houses only. In area 4, where temporal coverage of surveillance was sparser, infestation dropped from 17.7% to below target levels at 18 MPI, surged (8.7%) around 40 MPI, and then remained below target from 60 MPI onward, with a higher endpoint value (2.2%) than other areas. The prevalence of house infestation in domestic habitats closely followed the trajectories observed at house-compound level across areas (Fig 2B).

**Fig 3:**
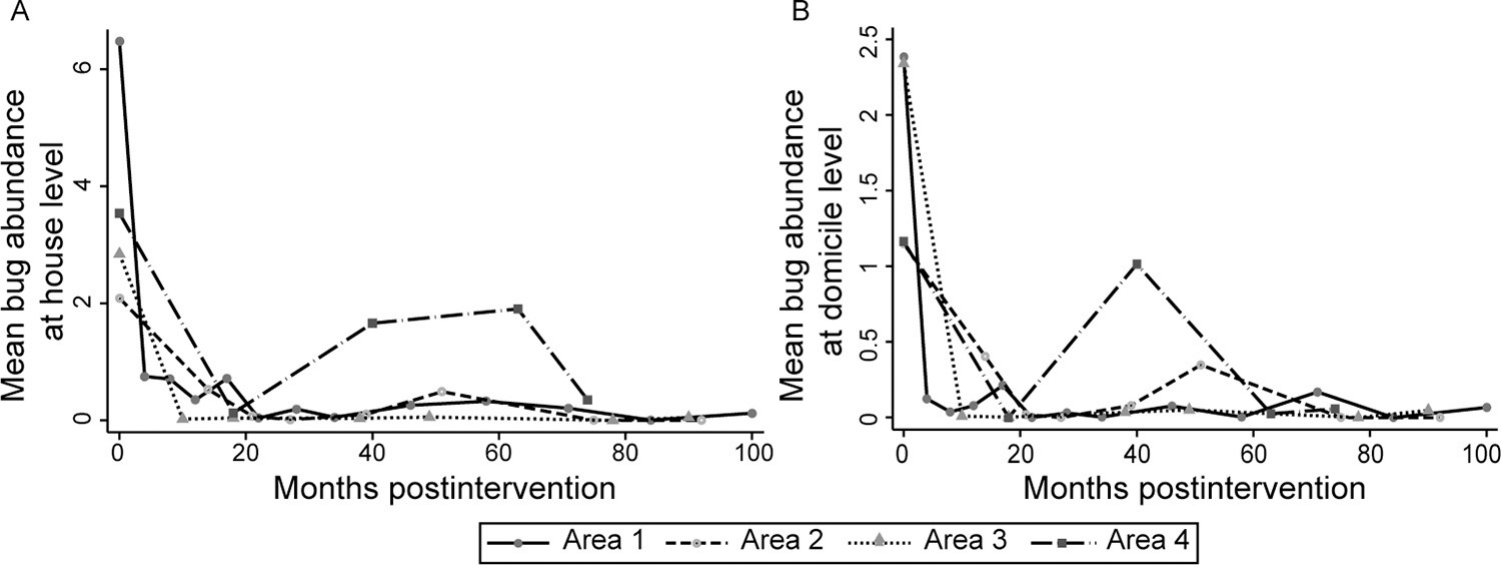
**Mean house abundance of *Triatoma infestans* at house compound-level (A) and in domiciles (B) according to months postintervention and operational area of Pampa del Indio, 2007–2016.** Vector abundance was determined by timed-manual searches. Includes all housing units inspected for infestation. Maps used base layers from Instituto Geográfico Nacional (Argentina) at: https://www.ign.gob.ar/NuestrasActividades/InformacionGeoespacial/CapasSIG. The maps were created in QGIS 2.18.11. based on the data collected within the scope of this study.

The baseline relative abundance of *T*. *infestans* at house-compound level averaged 3.9 (±0.4 SEM) bugs per unit effort and varied widely among areas: mean values in area 1 (6.5±0.8) doubled or tripled those in area 2 (2.1±0.6), 3 (2.8±0.5) and 4 (3.5±1.0) (Fig 3A). The time trajectories of mean bug abundance postintervention were similar to those displayed by house infestation indices across areas. Mean bug abundance at house-compound level remained depressed postintervention and ranged from 0 in areas 2 and 3 to 1.9 (±1.3) bugs per person-h in area 4. In domestic habitats, mean bug abundance plummeted over the first YPI and remained marginal over the advanced surveillance phase in area 1 (range, 0–0.22 bugs per unit effort), area 2 (range, 0–0.35), and area 3 (range, 0–0.05) (Fig 3B). Of the total catch of *T*. *infestans* across the follow-up period, 44.0% (3,158/7,172) occurred in domestic habitats. The mean ratios between domestic and peridomestic catches largely differed among areas, ranging from 0.44 (area 4) and 0.48 (area 1) to 1.75 (area 2) and 4.43 (area 3). The mean number of *T*. *infestans* foci per house compound at baseline (range, 0.18–0.59) also plummeted after the attack phase across areas albeit at a different pace, most evident in the contrast between areas 1 or 4 and 2 or 3.

### Spatial analysis

**Fig 4:**
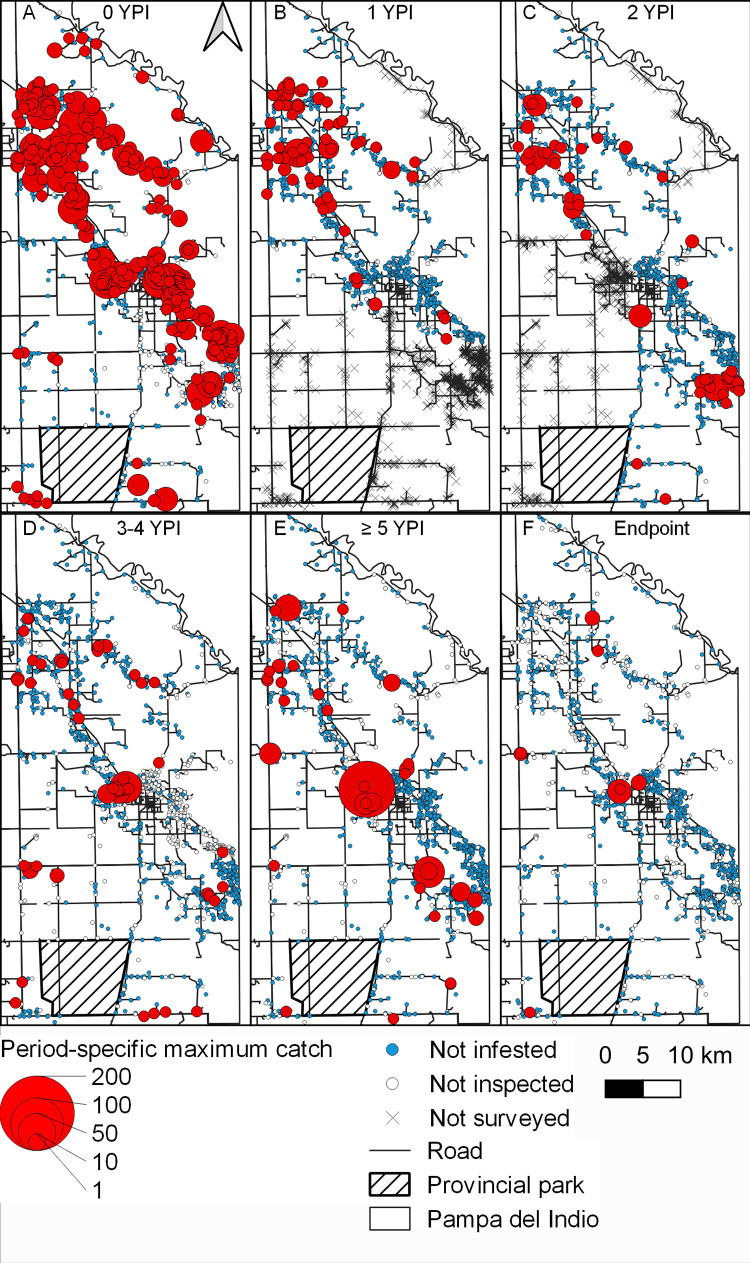
Distribution of maximum house abundance of *Triatoma infestans* per unit effort according to year postintervention with area-wide insecticide spraying, Pampa del Indio, 2007–2016. A: 0 YPI; B: 1 YPI; C: 2 YPI; D: 3–4 YPI; E: ≥5 YPI, and F: endpoint in 2016. Vector abundance was determined by timed-manual searches. Maps used base layers from Instituto Geográfico Nacional (Argentina) at: https://www.ign.gob.ar/NuestrasActividades/InformacionGeoespacial/CapasSIG. The maps were created in QGIS 2.18.11. based on the data collected within the scope of this study.

Fig 4 displays the time series of maximum triatomine abundance per house compound according to YPI across Pampa del Indio. Before the area-wide attack phase, triatomine abundance was widely distributed and apparently more concentrated across a densely populated NW–SE diagonal (Fig 4A). The borders with adjacent districts (all infested) also had large infestations at baseline. Control actions over the subsequent three years largely reduced the size of infestations and frequency of foci recorded at baseline, more so in the central and southeast sections (Fig 4B and 4D); the latter bordered with farmlands under intensified agriculture and no houses (Fig 1A and 1C). In addition to several foci related to prior aggregates, other foci detected over ≥5 YPI were on the borders with infested adjacent districts (Fig 4E and 4F).

Global spatial analysis revealed significant aggregation of triatomine abundance at 0 YPI within all radii considered barring 5,000 m, and at 1–3 YPI (4–39 MPI) within distances from 1,000 to 5,000 m (S3 Fig). Hence, aggregation of triatomine abundance occurred mainly at house compound or village levels (1,000–2,000 m) and disappeared after 3 YPI. For the extreme radii (400 and 5,000 m), aggregation levels expressed as L(r) values hardly varied across the study period. For intermediate radii (i.e., reflecting aggregation within or between villages), L(r) increased over time postintervention.

**Fig 5:**
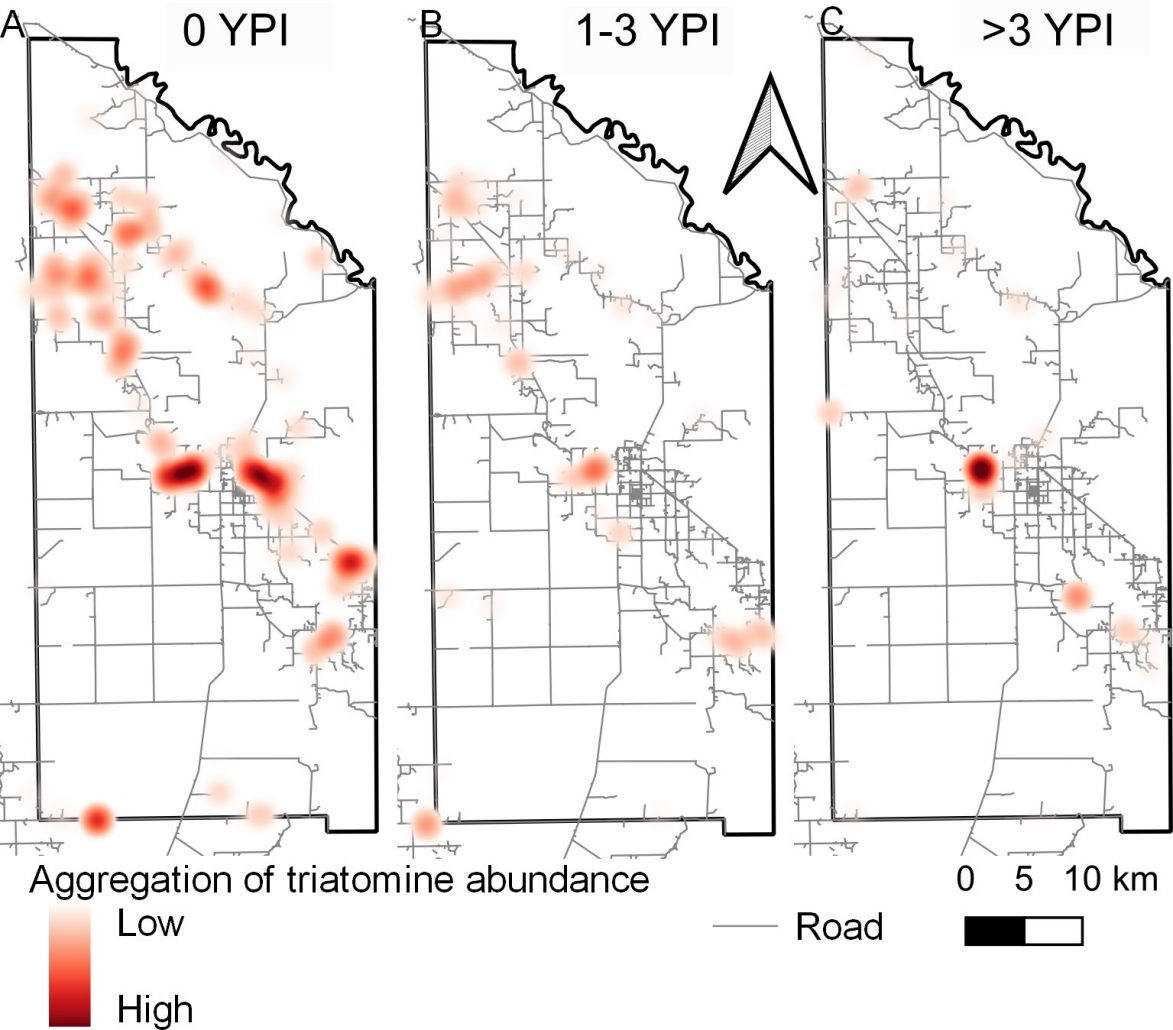
Heatmaps of maximum house abundance of *Triatoma infestans* per unit effort within 2,000 m according to year postintervention with area-wide insecticide spraying, Pampa del Indio, 2007–2016. A: 0 YPI; B: 1–3 YPI (4–39 MPI); C: >3 YPI. Vector abundance determined by timed-manual searches. Maps used base layers from Instituto Geográfico Nacional (Argentina) at: https://www.ign.gob.ar/NuestrasActividades/InformacionGeoespacial/CapasSIG. The maps were created in QGIS 2.18.11. based on the data collected within the scope of this study.

The time series of heatmaps shows a progressive reduction in the location, frequency and intensity of triatomine aggregation zones; Fig 5 illustrates the patterns observed at distances of 2,000 m (village level) over three periods. Three large clusters occurred preintervention (Fig 5A): the first one in the northwest across area 1; the second one occupying the core (around the main town and its sprawls), and the third one in the southeast section of the district. These clusters receded following spraying with pyrethroid and malathion (Fig 5B), but did not disappear altogether (Fig 5C). A closer view of the time series of maximum bug abundance in the core cluster shows that the hot spot nearly disappeared after the attack phase (Fig 4B) and reemerged later (Fig 4D–4F).

**Fig 6:**
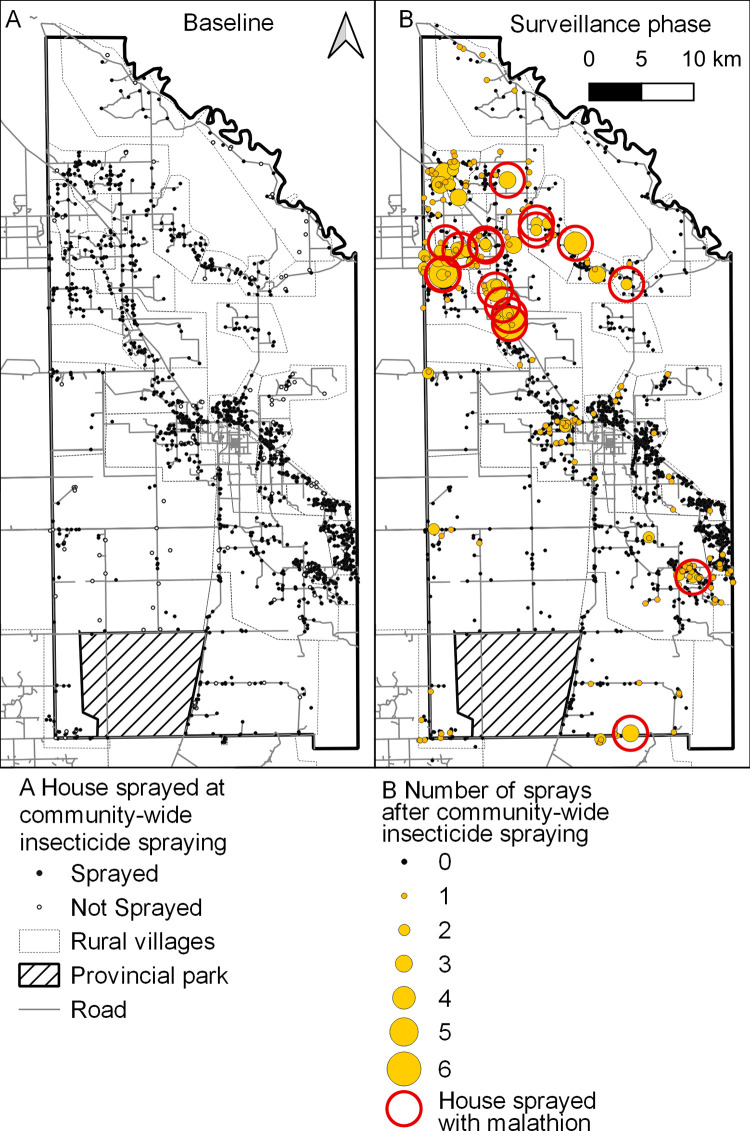
**Frequency distribution of house sprays with insecticide (pyrethroid and malathion) during the attack (A) and surveillance (B) phases across Pampa del Indio municipality, 2007–2016.** Maps used base layers from Instituto Geográfico Nacional (Argentina) at: https://www.ign.gob.ar/NuestrasActividades/InformacionGeoespacial/CapasSIG. The maps were created in QGIS 2.18.11. based on the data collected within the scope of this study.

House coverage with insecticide during the attack phase tended to be spatially homogeneous and virtually exhaustive across the district (Fig 6A). The cumulative frequency of house sprays with insecticide during the surveillance phase (nearly equal to how many times the house was observed infested by timed searches) was globally aggregated (S4 Fig): a large cluster of houses spread over area 1 and smaller clusters on the core and southeast border. The former overlapped with persistently infested houses that required the selective application of malathion (Fig 6B).

**Fig 7:**
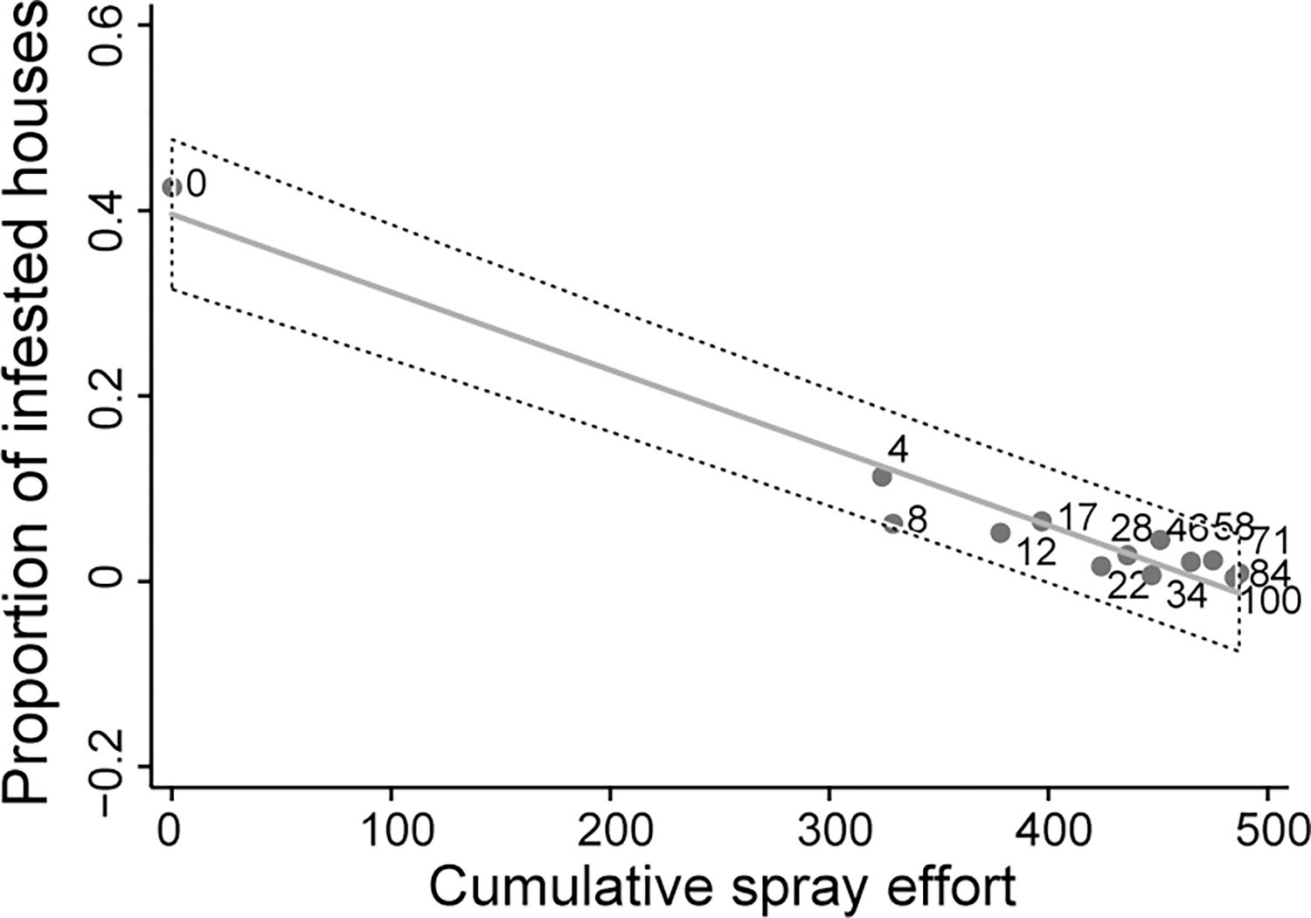
Linear ordinary regression of the mean prevalence of house infestation with *Triatoma infestans* at time *t* according to cumulative frequency of house sprays with insecticide at time *t*-1 in Pampa del Indio area 1. Includes all occupied or vacant housing units inspected for infestation. House infestation was determined by timed-manual searches. The dashed belt above and below each solid regression line represents a 95% confidence interval for individual residuals. Numbers next to the circles represent months postintervention.

Fig 7 illustrates the observed decline in mean prevalence of house infestation at time *t* as a function of cumulative control effort (quantified by the number of house sprays with insecticide at time *t*-1) in area 1 over time postintervention using ordinary linear regression. Mean infestation prevalence declined linearly and significantly with increasing cumulative spray effort (*a* = 0.39589 ± 0.24797; *b* = –0.00084 ± 0.00006, *adj*. *R*^*2*^ = 0.941, *n* = 13). Mean bug abundance displayed a similar strong relationship to cumulative control effort (*a* = 5.92462 ± 0.53780; *b* = –0.01306 ± 0.00131, *adj*. *R*^*2*^ = 0.892, *n* = 13).

### Pyrethroid resistance

**Fig 8:**
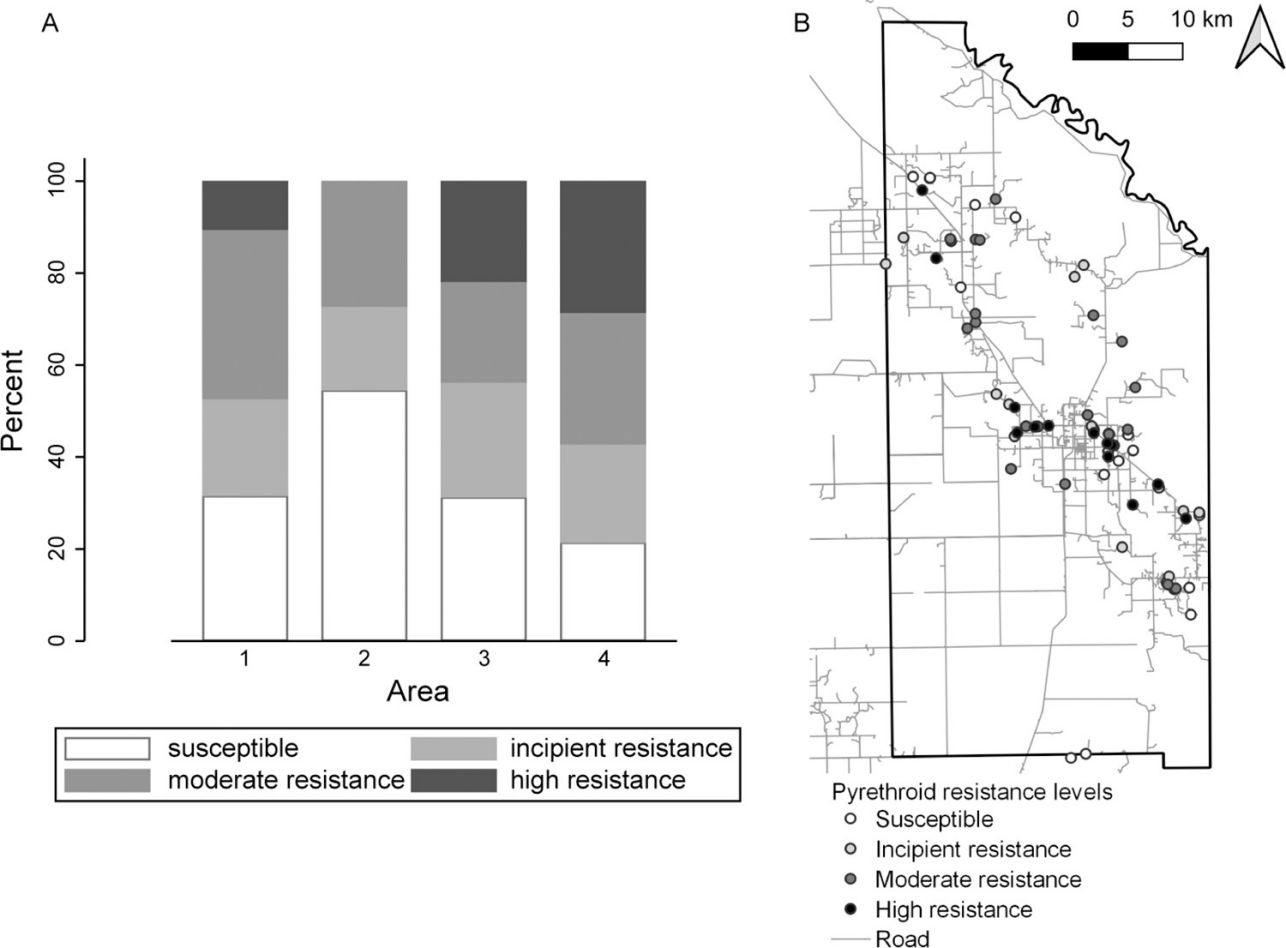
**Population distribution of pyrethroid resistance levels in *Triatoma infestans*, as determined by discriminant dose assays, by operational area (A) and across Pampa del Indio (B), 2007–2016.** Susceptible, > 90% bug mortality; incipient resistance, 76–90% mortality; moderate resistance, 45–75% mortality; and high resistance, <45% mortality. Numbers above bars indicate the number of triatomine populations (houses) tested. Maps used base layers from Instituto Geográfico Nacional (Argentina) at: https://www.ign.gob.ar/NuestrasActividades/InformacionGeoespacial/CapasSIG. The map was created in QGIS 2.18.11. based on the data collected within the scope of this study.

Of 76 *T*. *infestans* populations screened for pyrethroid resistance across the follow-up period, 61 (67%) expressed reduced mortality relative to the fully susceptible reference population (Fig 8A). Twenty-five (33%) triatomine populations were susceptible to pyrethroids. Resistance occurred at incipient levels (i.e., inducing 76–90% bug mortality) in 17 (22%) populations; at moderate levels (inducing 45–75% mortality) in 21 (28%) populations, and at high levels (<45% mortality) in 13 (17%) populations. Reduced bug mortality was detected across all areas both in Qom and creole households. Moderate or high pyrethroid resistance peaked in areas 4 (57%), 3 (44%) and 1 (47%). Resistance levels were not significantly associated with operational area (Fisher’s exact test, *P* = 0.66), with no obvious clustering across the district (Fig 8B), and surveillance phase period, categorized in two levels dictated by sample availability: up to the third postintervention survey, and from the fourth postintervention survey onwards (Fisher’s exact test, *P* = 0.09).

### Determinants of house infestation

Table 2 shows the association between house-level domestic and peridomestic infestation with *T*. *infestans* at baseline and over the advanced surveillance phase (>14 MPI) as determined by timed searches in each operational area. Before area-wide insecticide spraying, infestation was observed twice as often in domiciles only (174) than in peridomiciles only (91) of occupied or vacant houses inspected for triatomines. The area-adjusted relative odds for domestic infestation with *T*. *infestans* was 2.19 (95% confidence interval, 95% CI, 1.45–3.29) times greater when peridomiciles were simultaneously infested rather than not (CMH χ^2^ = 14.9, *df* = 1, *P* < 0.001). Significant associations were recorded in three of the four areas (i.e., excluding area 3), but there was not enough evidence of heterogeneity (χ^2^ = 5.59, *df* = 3, *P* = 0.13). During the advanced surveillance phase, domestic and peridomestic infestations adjusted for area were more strongly associated (OR = 14.74, 95% CI = 7.41–29.31, CMH χ^2^ = 104.1, *df* = 1, *P* < 0.001) than at baseline, with strong evidence of heterogeneity among areas (χ^2^ = 23.6, *df* = 3, *P* < 0.001) mostly related to area 3, where peridomestic structures were sparse.

**Table 2:** Association between concurrent domestic and peridomestic house infestation with *T*. *infestans* at baseline and over the advanced surveillance phase (>14 MPI) by operational area of Pampa del Indio. Includes occupied or vacant houses. House infestation was determined by timed-manual searches.

Intervention period	Area	Positive in domiciles only	Positive in peridomiciles only	Both positive	Both negative
Attack Phase	1	52	48	33	180
	2	15	8	3	154
	3	83	20	5	294
	4	24	15	7	214
	Total	174	91	48	842
Surveillance Phase	1	24	38	6	2,633
	2	15	9	1	2,095
	3	5	1	2	1,778
	4	16	13	2	611
	Total	60	61	11	7,117

House infestation with *T*. *infestans* determined by timed searches over the advanced surveillance phase was strongly and positively related to baseline house infestation status across areas (Table 3, top). Overall, 12.8% of baseline-positive houses were ever found infested subsequently, whereas 3.1% of baseline-negative houses were subsequently ever observed infested. The relative odds of a future house infestation was 3.75 (95% CI = 2.11–6.65) times greater when the house had been observed infested at baseline rather than when it had not (CMH χ^2^ = 23.6, *df* = 1, *P* < 0.001), with no significant heterogeneity among areas (χ^2^ = 0.70, *df* = 3, *P* = 0.87). Similarly, future house infestation increased highly significantly with baseline house infestation adjusted for household ethnicity (OR = 4.72, 95% CI = 2.77–8.05, CMH χ^2^ = 39.7, *df* = 1, *P* < 0.001), with no significant heterogeneity among areas (χ^2^ = 0.1, *df* = 3, *P* = 0.81) (Table 3, bottom).

**Table 3:** Association between house infestation with *T*. *infestans* at baseline and over the advanced surveillance phase (>14 MPI) by operational area of Pampa del Indio and according to household ethnicity. Includes occupied or vacant houses at baseline. House infestation was determined by timed-manual searches.

Factor	Level	Positive at baseline			Negative at baseline		
		Negative ever after	Positive ever after	% infested	Negative ever after	Positive ever after	% infested
Area	1	101	32	24.1	167	13	7.2
	2	22	4	15.4	146	8	5.2
	3	105	3	2.8	292	2	0.7
	4	45	1	2.2	211	3	1.4
	Total	273	40	12.8	816	26	3.1
Household ethnicity	Qom	147	10	6.4	391	5	1.3
	Creole	124	30	19.5	395	21	6.0
	Total	271	40	12.9	786	26	5.0

**Fig 9:**
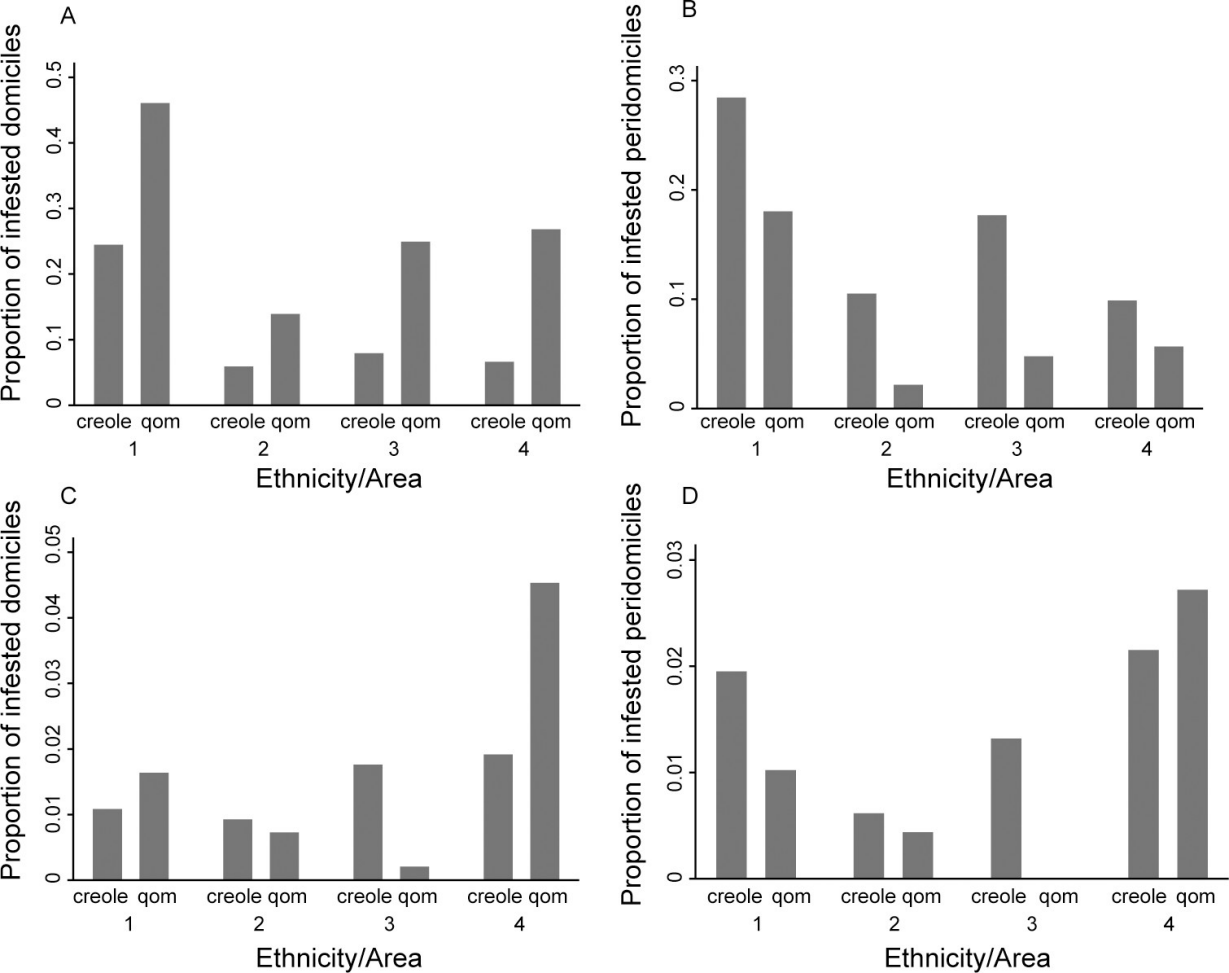
**Prevalence of house infestation with *Triatoma infestans* in domestic (A) and peridomestic (B) habitats at baseline and over the advanced surveillance phase (>14 MPI) (C, D, respectively) according to household ethnicity and operational area of Pampa del Indio, 2007–2016.** House infestation was determined by timed-manual searches.

Fig 9 shows the relationship between household ethnicity and house infestation with *T*. *infestans* in domestic and peridomestic habitats by area and intervention period. Before area-wide insecticide spraying, domestic infestation was consistently much greater in Qom households stratified by area (OR = 3.38, 95% CI = 2.21–5.16; CMH χ^2^ = 36.0, *df* = 1, *P* < 0.001; homogeneity χ^2^ = 1.95, *df* = 3, *P* = 0.6) (Fig 9A). Conversely, peridomestic infestation was highly significantly greater in creole households across areas (OR = 0.40, 95% CI = 0.24–0.65; CMH χ^2^ = 14.5, *df* = 1, *P* < 0.001; homogeneity χ^2^ = 3.37, *df* = 3, *P* = 0.34) (Fig 9B). A similar analysis over the advanced surveillance phase showed no significant effects of household ethnicity on domestic (OR = 1.14, 95% CI = 0.72–1.81; CMH χ^2^ = 0.31, *df* = 1, *P* = 0.57) and peridomestic infestation (OR = 0.61, 95% CI = 0.35–1.07; CMH χ^2^ = 3.1, *df* = 1, *P* = 0.08), although significantly heterogeneous effects were detected in both habitats across areas (homogeneity χ^2^ = 17.5 and 14.2, *df* = 3, *P* < 0.001 and *P* = 0.003, respectively) (Fig 9C and 9D).

When house-level infestation was assessed by timed searches, Qom households were significantly more frequently infested than creole ones across areas before area-wide insecticide spraying (OR = 1.57, 95% CI = 1.12–2.21; CMH χ^2^ = 6.8, *df* = 1, *P* = 0.009; homogeneity χ^2^ = 2.57, *df* = 3, *P* = 0.46), not over the advanced surveillance phase (OR = 0.82, 95% CI = 0.56–1.20; CMH χ^2^ = 1.03, *df* = 1, *P* = 0.31; homogeneity χ^2^ = 18.66, *df* = 3, *P* < 0.001). Similarly, house-level infestation assessed by householder triatomine collections at baseline returned significantly greater odds of infestation in Qom versus creole households (OR = 2.00, 95% CI = 1.17–3.42; CMH χ^2^ = 6.70, *df* = 1, *P* = 0.010; homogeneity χ^2^ = 2.3, *df* = 3, *P* = 0.51), and insignificant differences over >14 MPI (OR = 1.52, 95% CI = 0.87–2.67; CMH χ^2^ = 2.18, *df* = 1, *P* = 0.14; homogeneity χ^2^ = 3.63, *df* = 3, *P* = 0.16).

Zero-inflated negative binomial regression revealed that Qom households had a nearly six times greater domestic triatomine abundance than creole households across areas (IRR = 5.84; 95% CI, 2.94–11.60; Wald χ^2^ = 52.6, *df* = 4, *P* < 0.001), whereas the latter sustained significantly greater peridomestic triatomine numbers than Qom households (IRR = 0.16, 95% CI, 0.07–0.39; Wald χ^2^ = 91.5, *df* = 4, *P* < 0.001). Triatomine abundance at house-compound level did not significantly differ between Qom and creole households (IRR = 1.09, 95% CI, 0.60–1.97; Wald χ^2^ = 12.0, *df* = 4, *P* = 0.02).

## Discussion

The 9-year intervention program achieved the quasi-elimination of *T*. *infestans* at a district-wide scale in resource-constrained, remote rural settings through gradual scaling up of control actions and systematic vector surveillance-and-response within an adaptive management framework. Both the exploratory and baseline surveys had revealed high indices of domestic infestation and vector infection with *T*. *cruzi* indicating the occurrence of active transmission in human sleeping quarters [26,49,70]. The intervention program goals were met despite the presence of three major adversities: poor housing quality linked to chronic social deprivation, not restricted to indigenous households; persistent occurrence of *T*. *infestans* populations with high pyrethroid resistance in adjacent rural districts, and emergence of incipient-to-high levels of pyrethroid resistance in target populations of *T*. *infestans* leading to spatially aggregated control failures. Three factors that facilitated progress were the lower triatomine abundance levels in Pampa del Indio than in other rural sections of the dry Argentine Chaco under sporadic vector control; having two borders with no potential external sources of infestation, and elevated housing turnover combined with local household mobility in some district sections. Control operations met the initial target (house infestation <5%) in approximately one year across areas except in area 1 where pyrethroid resistance induced vector control failures. Low infestation levels were maintained over 2–5 YPI by means of surveillance and selective insecticide sprays, leading to rare, aggregated foci over 6–9 YPI, all of low density. These results reaffirm that conventional, high-quality triatomine control practices including adaptive responses to local specificities can be effective in rural communities of the Argentine Chaco despite major adversities. Such effectiveness was widely heterogeneous and implied increased operational costs.

The Pampa del Indio project provided valuable information on the quantity of surveillance-and-control efforts and amount of insecticide needed for reaching advanced levels of district-wide control of *T*. *infestans* and apparent interruption of vector-borne transmission. Field operations over nearly a decade roughly included 11,000 timed searches for triatomines in up to 2,300 registered houses and 1,800 insecticide treatments averaging 3.4 monodose units each, with only 18.0% of all treatments carried out after the one-cycle attack phase. Consequently, control efforts heavily leaned on vector surveillance-and-response rather than repeat, blanket insecticide application rounds. We adopted several decision criteria inspired on judicious application of pesticides, program sustainability and householder acceptance of interventions. Had the attack phase included a two-cycle area-wide spraying with insecticides, an additional 1,500 house sprays would have been implemented at the outset. The total cost of spraying a house with pyrethroid insecticide at a district-wide scale during the attack phase ranged between US$30 and 90 in the 1990s [54,71,72]. The numerous new houses appearing during the surveillance phase were not preemptively treated with insecticides regardless of infestation status (except in area 3 at 10 MPI) on the assumption that the risk of inadvertently carrying an infestation within Pampa del Indio at such times was marginal, as observed. When householders collected isolated specimens of *T*. *infestans* during the surveillance phase and concurrent timed searches failed to catch any, these houses were not sprayed with insecticides and continued under close surveillance. Similarly, the decision to forego insecticide application in most houses with *T*. *sordida* was based on several pieces of evidence suggesting this species was of minimal public health significance, if any. *Triatoma sordida* predominated in peridomestic habitats (~1,000 foci detected throughout), where it was strongly associated with chicken coops or nests, and failed to colonize domestic habitats regardless of insecticide spray status and absence of *T*. *infestans* [37,73]. *Triatoma sordida* had marginal or nil infection rates with *T*. *cruzi* [48,74] and displayed remarkable population recovery rates; flight-dispersing adult triatomines were frequently collected by householders [37,48]. Large-scale, long-term surveillance efforts of vector control programs in Brazil recorded similar patterns for *T*. *sordida* (e.g., [55]). In summary, indiscriminate house treatments would have required roughly a two to three times larger insecticide spray effort as that actually invested, and would have not averted vector control failures induced by pyrethroid resistance.

Target infestation levels were reached after one round of area-wide insecticide spraying and one or two annual vector surveys combined with selective treatments in three operational areas, whereas area 1 presented major challenges (see below). Mean house infestation prevalence or bug abundance declined linearly with lagged cumulative spray effort in area 1 (Fig 7), with most gains accrued at the initial stage of the program and diminishing returns from 2 YPI onwards. This pattern was also corroborated in other areas, with stronger diminishing returns in areas 2 and 3. How often should monitoring surveys and insecticide sprays be conducted depends on ultimate program goals (e.g., vector elimination vs control or transmission blockage) and widely variable factors, including local triatomine abundance and population recovery rates, the invasibility of domestic premises (determined by housing type and householder control practices), proximity to external sources of triatomines, and parasite transmission risks, weighted against the available resources. These multiple heterogeneities are better handled within an adaptive management framework than under fixed, blind recipes (e.g., blanket annual or biannual spray rounds) when the goal is sustained vector control or transmission blockage, not regional extinction of the target vector. Detailed cost-effectiveness analyses are needed to assess the broad implications of alternative control tactics adapted to specific scenarios [54].

District-wide surveys revealed that house infestation and triatomine abundance declined at different rates among areas despite using approximately the same set of supervised procedures aiming at universal coverage (i.e., spatial heterogeneity). Housing dynamics varied widely among areas and between Qom and creole households. The four areas differed in several sociodemographic aspects connected with triatomine abundance (e.g., house density, frequency of Qom and creole households, housing turnover and reported indoor insecticide use). Among them, household ethnicity was intimately linked to (and acted as a surrogate of) socioeconomic determinants of housing quality, domestic host availability, household size and crowding, peridomestic outhouses, and house occupancy [48,50]. House demolition was most frequent among Qom households whereas house vacancy predominated in creole households. Housing turnover was especially intense in some areas predominantly Qom (e.g., area 3) and usually implied destruction of the former house, relocation and reconstruction nearby. While these actions associated with local household mobility can be assumed to be mostly detrimental for house infestation and triatomine abundance before the attack phase, its net effects during the surveillance phase (when local infestation was rare) are less clear; they may range from marginal to substantial when precarious housing is combined with in-migration or return migration of household members from infested areas. In practice, few in-migrants from other potentially infested districts were registered during the surveillance phase in area 3 [48] and elsewhere, and return in-migrants mostly came from urban centers unlikely to be infested [50]. House occupancy in area 1 declined gradually after 5 YPI, and local peri-urban squatter settlements grew out of rural-to-urban migration of both creole and Qom households in search for better access to healthcare and educational facilities, safe water and electricity [61]. Economic instability and lack of local job opportunities fueled the out-migration of young people to large cities. Qom households were spatially aggregated in a few sections (partly linked to collective land ownership), and so were social vulnerability, house infestation and human infection with *T*. *cruzi* in area 3 [27,50]. Despite the end-to-end frequency of occupied houses remained nearly the same, substantial changes in housing status over time and space provided an added layer of complexity to vector surveillance-and-control operations.

Upscale district-wide integration of demographic information with program inputs and outputs uncovered several sources of spatial, biological and socioecological heterogeneity. These included the aggregation of house infestation and maximum bug abundance before and after area-wide interventions (Fig 5); the cumulative frequency of control efforts needed to reach a defined target; the spatial co-occurrence of persistent foci of *T*. *infestans* and pyrethroid resistance, and the localized high-density of Qom households. The sections with higher house infestation and triatomine abundance levels, all in area 1, harbored multiple foci after repeated pyrethroid applications; these sections mainly included creole households harboring multiple peridomestic habitats with domestic animals. The prevalence of human seropositivity for *T*. *cruzi* in area 1 (39.8%)[26] largely exceeded the seroprevalence observed in area 3 (29.0%)[27] and areas 2 and 4 combined (24.4%). Taken together, these detailed results support the outcomes and decisions derived from the district-wide exploratory survey, which identified area 1 as having larger infestation rates and higher transmission risk. Whether the emerging levels of pyrethroid resistance were causally linked to increasing triatomine abundance and human infection with *T*. *cruzi* is plausible and remains a matter for further research. Spatial heterogeneities in triatomine abundance and human infection have also surfaced in other triatomine species and settings (e.g., [54,75–78]). Coupled heterogeneities between social deprivation, triatomine abundance and human infection with *T*. *cruzi* imply that the local basic reproductive number (R_0_) of *T*. *cruzi* would increase proportionally to the strength of the correlation between factors and its standard deviation relative to the homogeneous mixing case [79].

Area 1 displayed distinctive patterns: 23% of baseline-infested houses treated with pyrethroid insecticide were again observed infested at 4 MPI, and most of them remained so after being re-treated with pyrethroids at increasing doses [45,47]. While none of these and subsequent control failures could be traced back to technical flaws or weather effects, a field trial corroborated the persistence of an enclosed population of *T*. *infestans* despite four pyrethroid applications over nearly a year [47]. Subsequent discriminant dose assays revealed the occurrence of pyrethroid-resistant foci (scored as incipient to moderate at that time) in zones with documented control failures, which ultimately justified the selective application of malathion [45,47,49]. The emergence of pyrethroid resistance in Pampa del Indio as of 2008 was unforeseen since resistance was known to occur mainly in northwest Argentina and Bolivia [29], and few pyrethroid-based spraying campaigns targeting *T*. *infestans* had previously been conducted in Chaco Province. The subsequent finding of highly resistant populations of *T*. *infestans* in a neighboring municipality (Castelli) as of late 2010 [46,80], within 50 km of Pampa del Indio, may signal the putative source of the pyrethroid-resistant triatomines in area 1 and the observed spatial patterns its propagation. *Triatoma infestans* has often been transported to distant places in the luggage of travelers, firewood and timber. With an estimated flight range of roughly 2.5 km (e.g., [81]), flying adults of *T*. *infestans* may invade the peripheral houses of Pampa del Indio from untreated houses in adjacent districts (e.g., Fig 5D–5F). Two district borders were heavily infested: 36.9% of houses on the south (including Tacuruzal and Pampa Bandera in 2014), and 57% on the west (Castelli in 2018) [82]. These impending threats posed minor obstacles to reach the target and sustain a control status once the initially persistent foci were extinguished.

Many of the initially persistent foci were gradually suppressed with successive pyrethroid applications despite the local occurrence of incipient or moderate pyrethroid resistance. Several factors may have contributed to this favorable outcome: resistance levels ranged mainly from nil to incipient or moderate; most foci had a lower mean bug abundance than elsewhere in the dry Argentine Chaco (e.g., [64]); the distribution of pyrethroid resistance phenotypes in the offspring of female *T*. *infestans* is not uniform [83]; pyrethroid-resistant *T*. *infestans* have lower fitness than its susceptible conspecifics [84], and environmental stochasticity affects the viability of small populations, more so when they are repeatedly exposed to toxicants and extreme temperatures.

Triatomine abundance displayed three evident clusters at district level (Fig 5), some of which had surfaced within individual areas 1–3 [45,48,49]. The identified aggregation spots gradually waned postintervention. The late surveillance phase (Fig 4E) displayed an unusually intense hotspot of *T*. *infestans* (a chicken coop in CY village, sprayed and dismantled subsequently) in area 4, where inter-survey periods were longer and surveillance more difficult. Given that previous surveys had shown no evidence of house infestation in the index house nor were its adjacent units infested, it might be a secondary focus derived from a preexisting infestation somewhere in the neighborhood (Fig 4D) or a case of passive transport (see below). The case in point illustrates the emergence of a discrete hot spot that may drive bug propagation in the interface between rural and peri-urban settlements [e.g., 61].

House-level domestic infestation was positively and highly significantly associated with concurrent peridomestic house infestation across areas and program phases. Peridomestic infestation usually exceeded domestic infestation with pyrethroid-susceptible *T*. *infestans* elsewhere in the dry Argentine Chaco (e.g., [28,85]), where peridomestic foci were less vulnerable to pyrethroid sprays than domestic foci [64,86]. Significant gene flow occurred between domestic and peridomestic populations of *T*. *infestans* in northwest Argentina [87]. These peridomestic foci frequently remain as sources of triatomines that reinvade domestic premises and eventually trigger transmission.

The relative odds of domestic infestation was nearly three times greater in Qom rather than creole households across Pampa del Indio areas, whereas peridomestic infestation was consistently greater among creole households. This remarkable crossover held both when house infestation was revealed by timed searches or by householder bug collections. Qom households usually had fewer peridomestic structures and domestic animals than creoles [58,70]. Thus, availability of suitable habitats for triatomines and whether they were domestic or peridomestic varied in connection with socioeconomic determinants and household ethnicity. Distinction between domestic and peridomestic habitats is of prime relevance from a transmission standpoint as virtually all human infections with *T*. *cruzi* are acquired in domestic areas, which concentrated vector infection and human-triatomine contacts [88,89]. In Pampa del Indio, Qom households had substantially greater domestic infestation and triatomine abundance than creole households across areas and program phases, as was the unadjusted seroprevalence of human infection in areas 1 and 3 at baseline [26,27].

The strong relationship between postintervention and preintervention house infestation status across areas suggests that most postintervention findings of *T*. *infestans* were residual or persistent foci (i.e., sites where insecticide treatments failed to suppress local triatomines). Residual foci may originate from pyrethroid resistance, complex construction features, and technical flaws such as inadequate spray coverage [28]. Wing geometric morphometry [90] and microsatellite markers supported that most *T*. *infestans* collected over the first YPI in area 1 were the offspring or survivors of preintervention insects (vs immigrants from external sources), but microsatellite markers further revealed a few immigrant insects from unaccounted sources [91]. Residents of recently infested houses reported events of passive or active bug transport between peri-urban and rural areas and between neighboring districts (e.g., [48,49,61], as in the southern and northwestern borders where some villages sprawled into infested neighboring districts (Fig 1A). As local sylvatic foci of *T*. *infestans* have not been detected so far [92,93], the relative importance of external sources (reintroductions) most likely increased as the early residual foci were suppressed and the surveillance phase progressed.

The positive association between postintervention and preintervention house infestation or abundance of *T*. *infestans* appears to be generalized throughout the Argentine Chaco (e.g., [45,86,94] and in Arequipa city (Peru), where nonparticipation in insecticidal campaigns was a key factor explaining residual infestation [95], unlike in Pampa del Indio. The spatial clusters of house infestation were virtually unaffected by spraying with pyrethroids in the southern (dry) Argentine Chaco [85]. Similarly, in Cochabamba (Bolivia), the prevalence of district-wide house infestation was autocorrelated despite recurrent insecticide spraying, suggesting persistent foci were implicated [56]. In Jutiapa (Guatemala), postintervention house infestation with *Triatoma dimidiata* was positively related to its preintervention infestation status [78]. These empirical observations across settings and species strongly suggest that the effectiveness of residual spraying with pyrethroids is modified by preintervention house infestation status and triatomine abundance. Hence, such valuable information may assist in identifying high-risk households subject to increased risks of future infestation and eventual transmission.

Key to the interpretation of current study findings is the fact that most of the evidence on house infestation is based on the outcome of timed-manual searches with a dislodging spray, with ancillary results provided by partial use of other vector survey methods. The conditional probability of detecting a house infestation with *T*. *infestans* using timed-manual searches both in the exploratory and baseline surveys was 0.8. However, triatomine removal by timed searches conducted shortly apart may have reduced bug catch and detection probabilities on subsequent occasions [96], while the time gap between surveys in areas 2–4 allowed new invasions. Similarly, apparent detectability of timed searches at baseline (relative to the outcome of multiple detection methods and occasions) ranged from 61.4% to 88.0%, whereas in other studies timed searches detected from 28% to 75% of house infestations ascertained through various procedures (e.g., [82,95,97–100]). These results attest to the limited detectability of timed searches regardless of whether a pyrethroid-based aerosol is used to flush out triatomines from their refuges–an advantage mostly lost when insects are highly resistant to pyrethroids [101]. Nonetheless, detectability depends on multiple factors that are hard to standardize: technicians’ search skills and background experience; whether the habitat structure allows an in-depth probe for triatomines; local bug abundance, search time and temperature [97,99]. These multiple dependencies affect the precision of infestation and catch-per-unit-effort indices, which at best bear some proportionality to local bug abundance [102] above an undefined threshold. Despite its multiple limitations including cost, timed-manual searches have paradoxically been the reference in successful large-scale elimination programs of *T*. *infestans* and *Rhodnius prolixus* (e.g., [16]) and is the recommended standard [103]. Perhaps its shortcomings were compensated for by community-based surveillance and frequent re-treatment of houses.

In practice, the apparent prevalence of house infestation measured by timed searches returns a biased measure of true prevalence, which in principle could be corrected using an independent estimate of detectability or sensitivity [67,68]. The lack of a “gold standard” method hampers the estimation of detectability, a limitation sometimes addressed by using multiple detection methods or search occasions. Detectability estimates are further reduced when low-density infestations missed by timed searches are suppressed by a subsequent residual insecticide treatment intended to reveal triatomine presence through its irritant and knock-down effects [95]. Conversely, when individual habitats or houses are repeatedly searched for triatomines over a time period, the probability of obtaining a “false negative” outcome across standardized searches and occasions can be minimized. Assuming that an individual timed search has a constant probability of detecting triatomines (e.g., detectability = 0.60) at each of three independent search occasions in which site infestation status remains constant, the probability of ever detecting an underlying site infestation across occasions is 0.936 (i.e., probability of a false negative outcome = 0.064), an acceptable error for the specified goals. For an invasive species in suitable habitats such as *T*. *infestans*, local bug density is more likely to grow over time and thus detectability is expected to increase between spaced occasions and the false-negative rate decrease. Even if timed searches return biased estimates of house infestation or triatomine abundance, they would still provide an internally valid measure of relative change if bias (and standardized procedures) remained approximately constant over time [67]–a key issue rarely verified. This would fit the purpose of a monitoring program whose goal is to detect defined changes or trends in house infestation prevalence or triatomine abundance at some aggregate spatial level (area or village clusters). Comparison of entomological and parasitological outcomes also illuminate the actual value of classic indices (e.g., insect catch per unit effort) linked to vectorial capacity [104]. In Pampa del Indio, the apparent interruption of vector-borne transmission to humans [52] further supports that the underlying (true) domestic infestation levels over the prolonged follow-up were very low or low and below a putative, unobserved transmission threshold.

For decision making at individual house level (i.e., whether to spray a house with insecticide after a one-off search for triatomines), the outcome of a single timed-manual search has limited validity and will miss many foci, especially during the surveillance phase, when low-density infestations prevail, and when technical details are neglected (e.g., skilled personnel, search time, sample sizes). The implications are even more serious when point estimates of house infestation devoid of a confidence interval are used for policy making and district-wide risk assessment for certification goals. In the absence of cost-effective methods for large-scale triatomine surveillance, combining householders’ involvement and targeted timed searches conducted by skilled personnel in high-risk zones offers a way forward to avoid overconfidence. Other options include insecticide knock-down collections for ascertaining house infestation status and sensitive triatomine sensing devices, if and when available. In our study, householder-based surveillance provided an independent check of house infestation status; frequently detected triatomine invasion events, and defeated or equaled the output of concurrent timed searches in domiciles, not in peridomiciles, over the early surveillance phase [45,48,105]. Local Qom households returned triatomines as frequently as creoles or more, refuting the notion that indigenous people take a complacent stance toward house infestation. Householder-based surveillance articulated with local primary healthcare posts would largely reduce the operating costs of surveys involving vector control personnel brought in from distant locations. Effective community-based triatomine surveillance demands continuity, stimulating participation and providing timely control responses [99,106–108].

Our intervention program had some limitations linked to the realities of vector control in remote rural areas. The timing of the attack phase and subsequent vector surveys across areas was partly determined by the pace of scaling up operations, limited resource availability (e.g., personnel, vehicles, insecticide), and accessibility to the villages. Wet years with excess rainfall interfered with field operations, prolonged them, impeded access to some rural sections, and increased costs. Consequently, evaluations and control actions were conducted asynchronically; the district-wide maps offer the best available approximation to average estimates of various metrics for defined time periods. As house infestation dropped to low levels, the frequency of monitoring surveys were gradually reduced and house infestation data became sparser (e.g., area 4). Whether incipient housing improvement (mainly unfolding when infestations had become rare), variable rates of house occupancy among areas, and householder use of insecticide indoors contributed to the effectiveness of surveillance-and-response procedures remains for focused research.

### Implications for vector control

This intervention program demonstrates the feasibility of achieving advanced stages in the process of elimination of *T*. *infestans* in hyperendemic settings of the Argentine Chaco through systematic, professional application of conventional vector control procedures at a district-wide scale within an adaptive management framework. This is unlike the situation in sections of the dry Argentine Chaco, where sustained surveillance-and-response largely reduced domestic infestations with pyrethroid-susceptible *T*. *infestans* and interrupted transmission without ever reaching the low house infestation levels herein recorded [88]. While the amount of insecticide spray effort over the surveillance phase was limited (18% of those invested in a one-cycle attack phase), it was spatially and temporally heterogeneous and dependent of recurrent house inspections.

The emergence of pyrethroid resistance jeopardized initial vector elimination efforts, mostly because of delays in problem diagnosis and the paucity of safe, acceptable options for suppressing pyrethroid-resistant triatomine populations [29,80]. Rapid detection of pyrethroid resistance using impregnated filter papers [109] or molecular tools [110] at the outset of operations will reduce control efforts and save resources. However, new insecticides and formulations that can cope with peridomestic triatomine populations and pyrethroid-resistant foci in a cost-effective way are needed. Whether alpha-cypermethrin remains a viable option [111] for pyrethroid-resistant triatomine populations is unclear. Other options include long-lasting ectoparasiticides administered to dogs (e.g., fluralaner), which substantially reduced site abundance of pyrethroid-resistant *T*. *infestans* populations, bug infection and feeding contact with humans in a small field trial [112,113]. Pyrethroid-resistant populations of *T*. *infestans* persist in sections of the Gran Chaco and should be targeted for prompt elimination to prevent further spread and additional treatment costs [28]. Such impending threats to vulnerable communities underscore the need of sustained vector surveillance-and-response.

A key question faced by Chagas disease control programs is whether the low-density infestations recorded after district-wide insecticide spraying are indicative of human infection risks with *T*. *cruzi*. Here, the strong, negative relationship between insecticide spraying effort and domestic house infestation or abundance was consistent with the outcomes of serosurveys showing no evidence of *T*. *cruzi* transmission to the local human population and a marginal force of infection in dogs, possibly related to other transmission pathways [52]. The endpoint estimate of apparent prevalence of house infestation (0.66%) was compatible with levels associated with transmission interruption (1%) in Brazil [16]. The 95% confidence interval for endpoint domestic infestation prevalence (0.09–0.84%) also contained the target level (0.1%) of intradomestic infestation indices required for certification purposes [103]. More quantitative evidence on these putative transmission thresholds and whether these are adequate for the Gran Chaco are needed.

The project revealed multiple coupled heterogeneities (spatial, sociodemographic and biological) that reflect large socioeconomic inequalities, hamper control efforts, and provide opportunities for cost-effective, targeted control actions focusing on the most affected population subgroups or village clusters. Strong clustering of house infestation, pyrethroid resistance and sociodemographic determinants call out for an adaptive strategy rather than fixed, homogeneously effective recipes. Regional coordination of vector control efforts will decrease the risk of reintroductions from neighboring infested districts. The strong relationships between household ethnicity and domestic triatomine abundance were partly or fully verified across areas, and both were linked directly to social vulnerability and human infection with *T*. *cruzi* in area 3 [27,50]. In the light of a chronic housing debt and local job scarcity, this intervention program combined with etiologic treatment of infected children minimized current exposure and future Chagas disease burden [52,62] but was not meant to address or modify the underlying social determinants of health. Healthy housing and rural development policies may contribute substantially to the sustainable control of Chagas disease and other NTDs in the Gran Chaco.

## Supporting information

S1 Text Checklist of STROBE recommendations for observational studies. (DOCX)

S1 Fig Status of registered housing units as occupied (A), newly built (B), vacant (C) and demolished (D) over months postintervention by operational area of Pampa del Indio, 2007–2016.
(TIF)

S2 Fig Frequency of house treatments with insecticide during the attack and surveillance phases by operational area of Pampa del Indio (A) and household ethnicity (B), 2007–2016.
(TIF)

S3 Fig Global spatial analysis of house-level relative abundance of *Triatoma infestans* for radial distances of 400, 1,000, 2,000 and 5,000 m (A–D, respectively) according to months postintervention with area-wide insecticide spraying, Pampa del Indio. The observed statistics L(r) are shown as filled circles; the expected values under the null model as black lines, and confidence envelopes as grey lines.
(TIF)

S4 Fig **Global spatial analysis (A) of the number of house sprays with insecticides during the surveillance phase for radial distances up to 7,500 m and heatmap (B) for 2,000 m, Pampa del Indio.** Maps used base layers from Instituto Geográfico Nacional (Argentina) at: https://www.ign.gob.ar/NuestrasActividades/InformacionGeoespacial/CapasSIG. The map was created in QGIS 2.18.11. based on the data collected within the scope of this study. The observed statistic L(r) is shown as a red line; the expected values under the null model as black lines, and the 95% confidence envelope as a grey area.
(TIF)

S1 Table Housing demography and coverage of house infestation surveys and insecticide spraying by operational area of Pampa del Indio over 2007–2016. (DOCX)

S2 Table Individual house-level data including housing status, household ethnicity, infestation metrics and insecticide treatment in Pampa del Indio over 2007–2016. (XLS)

S3 Table Individual house data including infestation metrics at the pilot and baseline surveys in Pampa del Indio over 2007–2016. (XLS)

S4 Table Individual bug population data showing the outcome of dose discriminant assays for pyrethroid resistance in Pampa del Indio over 2007–2016. (XLS)
