# Supplementary material for: The Pampa del Indio project: District-wide quasi-elimination of Triatoma infestans after a 9-year intervention program in the Argentine Chaco
Source: PLoS Negl Trop Dis. 2023 Apr 24;17(4):e0011252. doi: 10.1371/journal.pntd.0011252 (PMC10159358; doi:10.1371/journal.pntd.0011252)
Supplement: S1 Text — (DOCX) [file pntd.0011252.s001.docx]

STROBE Statement—checklist of items that should be included in reports of observational studies

|  | | Item No. | | Recommendation | Page  No. | | Relevant text from manuscript |
| --- | --- | --- | --- | --- | --- | --- | --- |
| **Title and abstract** | | 1 | | (*a*) Indicate the study’s design with a commonly used term in the title or the abstract | 2 | | Lines 24- 27 |
|  |  |  |  | (*b*) Provide in the abstract an informative and balanced summary of what was done and what was found | 2 | | Lines 34-51 |
| Introduction | | | | | | |  |
| Background/rationale | | 2 | | Explain the scientific background and rationale for the investigation being reported | 4-7 | | Lines 64-174 |
| Objectives | | 3 | | State specific objectives, including any prespecified hypotheses | 7-8 | | Lines 175-203 |
| Methods | | | | | | |  |
| Study design | | 4 | | Present key elements of study design early in the paper | 11-12 | | Lines 294-321 |
| Setting | | 5 | | Describe the setting, locations, and relevant dates, including periods of recruitment, exposure, follow-up, and data collection | 8-10  12-17 | | Lines 212-272  Lines 323-472  Fig 1  S1 Table |
| Participants | | 6 | | (*a*) *Cohort study*—Give the eligibility criteria, and the sources and methods of selection of participants. Describe methods of follow-up  *Case-control study*—Give the eligibility criteria, and the sources and methods of case ascertainment and control selection. Give the rationale for the choice of cases and controls  *Cross-sectional study*—Give the eligibility criteria, and the sources and methods of selection of participants | 12  12-16 | | Lines 312-321  Lines 323-457 |
|  |  |  |  | (*b*) *Cohort study*—For matched studies, give matching criteria and number of exposed and unexposed  *Case-control study*—For matched studies, give matching criteria and the number of controls per case |  | |  |
| Variables | | 7 | | Clearly define all outcomes, exposures, predictors, potential confounders, and effect modifiers. Give diagnostic criteria, if applicable | 10-11  14  15-16 | | Lines 274-292  Lines 371-398  Lines 428-434 |
| Data sources/ measurement | | 8* | | For each variable of interest, give sources of data and details of methods of assessment (measurement). Describe comparability of assessment methods if there is more than one group | 12 -16  16-17 | | Lines 312-434  Lines 458-472 |
| Bias | | 9 | | Describe any efforts to address potential sources of bias | 14  15 | | Lines 371-379  Lines 404-405 |
| Study size | | 10 | | Explain how the study size was arrived at | 19 | | Lines 538-546  S1 Table and S1 Fig |
| Quantitative variables | 11 | | Explain how quantitative variables were handled in the analyses. If applicable, describe which groupings were chosen and why | | 17-19 | Lines 474-534 | |
| Statistical methods | 12 | | (*a*) Describe all statistical methods, including those used to control for confounding | | 17-19 | Lines 474-521 | |
|  |  |  | (*b*) Describe any methods used to examine subgroups and interactions | | 18 | Lines 509-511 | |
|  |  |  | (*c*) Explain how missing data were addressed | | 14 | Lines 389-390 | |
|  |  |  | (*d*) *Cohort study*—If applicable, explain how loss to follow-up was addressed  *Case-control study*—If applicable, explain how matching of cases and controls was addressed  *Cross-sectional study*—If applicable, describe analytical methods taking account of sampling strategy | |  | S1 Fig and S1 Table | |
|  |  |  | (*e*) Describe any sensitivity analyses | | 18-19 | Lines 488-498 | |
| Results | | | | | | | |
| Participants | 13* | | (a) Report numbers of individuals at each stage of study—eg numbers potentially eligible, examined for eligibility, confirmed eligible, included in the study, completing follow-up, and analysed | | 19  21 | Lines 538-547  S1 Fig and S1 Table  Lines 593-601 | |
|  |  |  | (b) Give reasons for non-participation at each stage | | 22 | S1 Table  Lines 621-623 | |
|  |  |  | (c) Consider use of a flow diagram | |  |  | |
| Descriptive data | 14* | | (a) Give characteristics of study participants (eg demographic, clinical, social) and information on exposures and potential confounders | | 19    22 | Lines 538-547  Fig 1  S1 Fig and S1 Table  Lines 593-601 | |
|  |  |  | (b) Indicate number of participants with missing data for each variable of interest | | 22 | Lines 606-608 | |
|  |  |  | (c) *Cohort study*—Summarise follow-up time (eg, average and total amount) | |  | S1 Fig and S1 Table  S2 Fig | |
| Outcome data | 15* | | *Cohort study*—Report numbers of outcome events or summary measures over time | | 19-24 | Lines 537-676  Table 1  Fig 2,3,4,5,6,7 | |
|  |  |  | *Case-control study—*Report numbers in each exposure category, or summary measures of exposure | |  |  | |
|  |  |  | *Cross-sectional study—*Report numbers of outcome events or summary measures | |  |  | |
| Main results | 16 | | (*a*) Give unadjusted estimates and, if applicable, confounder-adjusted estimates and their precision (eg, 95% confidence interval). Make clear which confounders were adjusted for and why they were  included | | 22-24  24-30 | Lines 618-670  Fig 2, Table 2, Table 3  Lines 679-837 | |
|  |  |  | (*b*) Report category boundaries when continuous variables were categorized | |  |  | |
|  |  |  | (*c*) If relevant, consider translating estimates of relative risk into absolute risk for a meaningful time period | |  |  | |

Continued on next page

| Other analyses | 17 | Report other analyses done—eg analyses of subgroups and interactions, and sensitivity analyses | 26-27  27-30 | Lines 746-758  Fig 8  Lines 766-837  Fig 9  S3 Fig and S4 Fig |
| --- | --- | --- | --- | --- |
| Discussion | | | | |
| Key results | 18 | Summarise key results with reference to study objectives | 30-31 | Lines 840-861 |
| Limitations | 19 | Discuss limitations of the study, taking into account sources of potential bias or imprecision. Discuss both direction and magnitude of any potential bias | 40-41  37-38 | Lines 1123-1135  Lines 1051-1073 |
| Interpretation | 20 | Give a cautious overall interpretation of results considering objectives, limitations, multiplicity of analyses, results from similar studies, and other relevant evidence | 30-39 | Lines 840-1122 |
| Generalisability | 21 | Discuss the generalisability (external validity) of the study results | 37  40-42 | Lines 1042-1050  Lines 1137-1188 |
| Other information | |  | | |
| Funding | 22 | Give the source of funding and the role of the funders for the present study and, if applicable, for the original study on which the present article is based |  | Online subsmission system |

*Give information separately for cases and controls in case-control studies and, if applicable, for exposed and unexposed groups in cohort and cross-sectional studies.

**Note:** An Explanation and Elaboration article discusses each checklist item and gives methodological background and published examples of transparent reporting. The STROBE checklist is best used in conjunction with this article (freely available on the Web sites of PLoS Medicine at http://www.plosmedicine.org/, Annals of Internal Medicine at http://www.annals.org/, and Epidemiology at http://www.epidem.com/). Information on the STROBE Initiative is available at www.strobe-statement.org.
