# Supplementary figures and images for: The Pampa del Indio project: District-wide quasi-elimination of Triatoma infestans after a 9-year intervention program in the Argentine Chaco
Source: PLoS Negl Trop Dis. 2023 Apr 24;17(4):e0011252. doi: 10.1371/journal.pntd.0011252 (PMC10159358; doi:10.1371/journal.pntd.0011252)

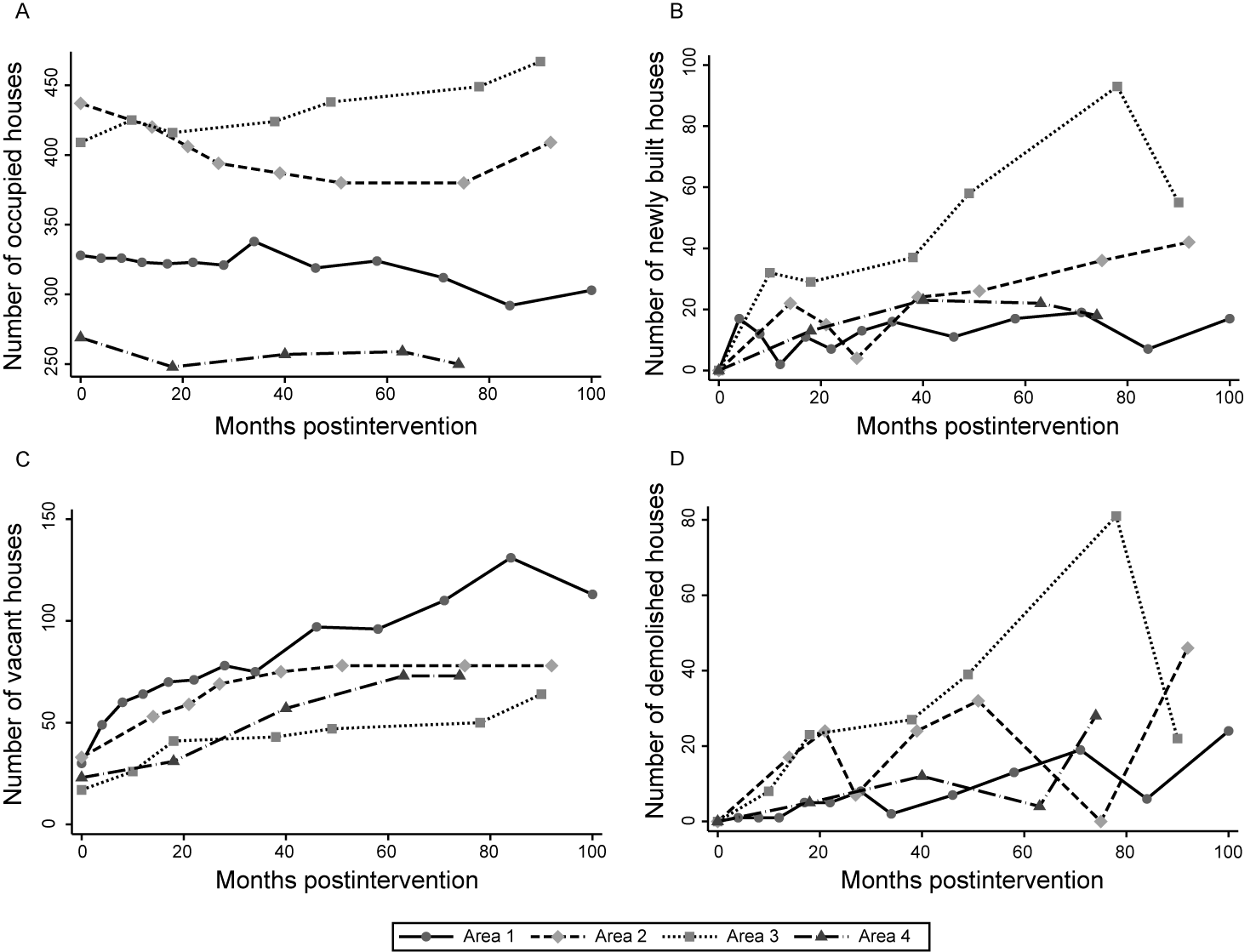

Supplement: S1 Fig — Status of registered housing units as occupied (A), newly built (B), vacant (C) and demolished (D) over months postintervention by operational area of Pampa del Indio, 2007–2016. (TIF) [file pntd.0011252.s002.tif]

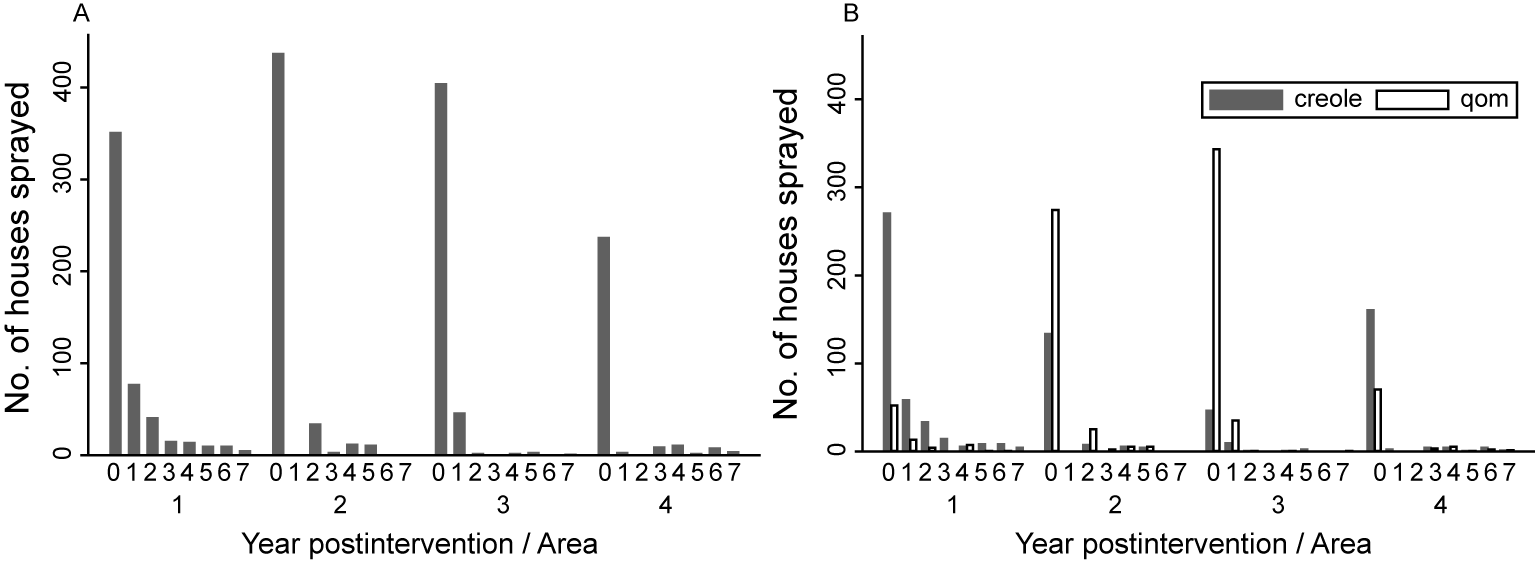

Supplement: S2 Fig — Frequency of house treatments with insecticide during the attack and surveillance phases by operational area of Pampa del Indio (A) and household ethnicity (B), 2007–2016. (TIF) [file pntd.0011252.s003.tif]

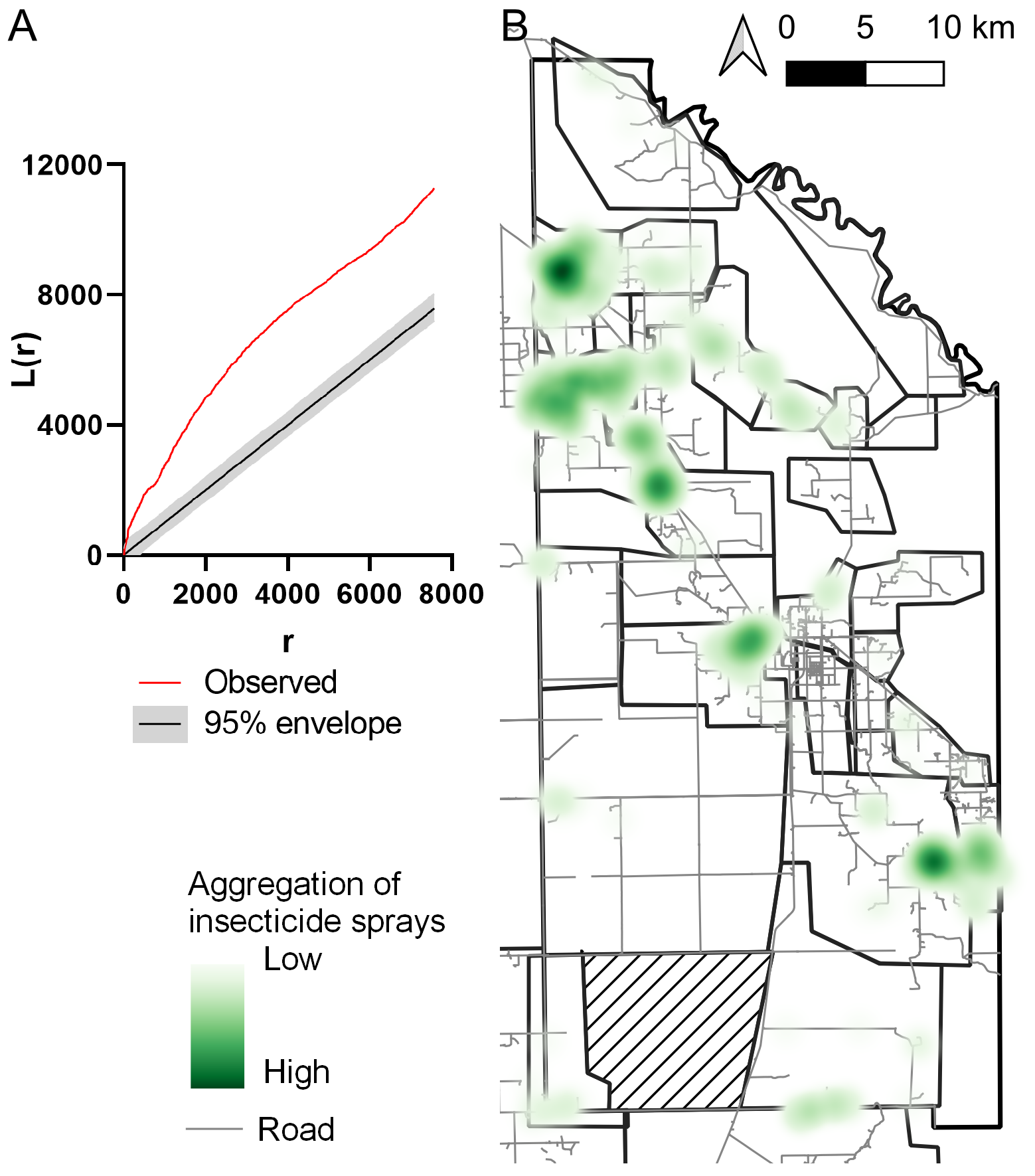

Supplement: S4 Fig — Global spatial analysis (A) of the number of house sprays with insecticides during the surveillance phase for radial distances up to 7,500 m and heatmap (B) for 2,000 m, Pampa del Indio. Maps used base layers from Instituto Geográfico Nacional (Argentina) at: https://www.ign.gob.ar/NuestrasActividades/InformacionGeoespacial/CapasSIG. The map was created in QGIS 2.18.11. based on the data collected within the scope of this study. The observed statistic L(r) is shown as a red line; the expected values under the null model as black lines, and the 95% confidence envelope as a grey area. (TIF) [file pntd.0011252.s005.tif]
