# Supplementary material for: The Pampa del Indio project: District-wide quasi-elimination of Triatoma infestans after a 9-year intervention program in the Argentine Chaco
Source: PLoS Negl Trop Dis. 2023 Apr 24;17(4):e0011252. doi: 10.1371/journal.pntd.0011252 (PMC10159358; doi:10.1371/journal.pntd.0011252)
Supplement: S1 Table — (DOCX) [file pntd.0011252.s006.docx]

**S1 Table. Housing demography and coverage of house infestation surveys and insecticide spraying by operational area of Pampa del Indio over 2007-2016.**

| Area | Months post-intervention | Date of vector survey (month-year) | Occupied | Vacant | Demolished | New | Inspected for triatomines | Sprayed with insecticides | Insecticide | Pyrethroid dose^a^ |
| --- | --- | --- | --- | --- | --- | --- | --- | --- | --- | --- |
| 1 | 0 | oct-07 | 328 | 30 | 0 | 0 | 324 | 387 | SC deltamethrin | Simple |
|  | 4 | apr-08 | 326 | 49 | 1 | 17 | 372 | 6^b^ | SC deltamethrin | Simple |
|  | 8 | aug-08 | 326 | 60 | 1 | 12 | 373 | 52 | SC deltamethrin | Simple or double |
|  | 12 | dec-08 | 323 | 64 | 1 | 2 | 344 | 19 | SC beta-cypermethrin | Simple or double |
|  | 17 | may-09 | 318 | 70 | 5 | 7 | 358 | 29 | SC beta-cypermethrin | Double |
|  | 22 | oct-09 | 319 | 71 | 5 | 7 | 367 | 12 | Malathion | Simple |
|  | 28 | apr-10 | 318 | 77 | 8 | 13 | 359 | 11 | Malathion | Simple |
|  | 34 | oct-10 | 335 | 74 | 2 | 16 | 314 | 4 | SC deltamethrin | Double |
|  | 46 | oct-11 | 316 | 96 | 7 | 11 | 246 | 14 | SC deltamethrin | Simple |
|  | 58 | oct-12 | 320 | 95 | 13 | 16 | 286 | 10 | SC deltamethrin | Simple |
|  | 71 | nov-13 | 308 | 109 | 19 | 19 | 315 | 10 | SC deltamethrin | Simple |
|  | 84 | dec-14 | 289 | 130 | 5 | 7 | 242 | 2 | SC deltamethrin | Simple |
|  | 100 | apr-16 | 300 | 112 | 24 | 17 | 238 | 3 | SC beta-cypermethrin | Simple (domi), double (peri) |
| 2 | 0 | aug-08^c^ | 437 | 33 | 0 | 0 | 180 | 460 | SC deltamethrin | Simple |
|  | 14 | aug-09 | 420 | 53 | 17 | 22 | 404 | 24 | SC beta-cypermethrin or SC deltamethrin | Simple |
|  | 21 | may-10 | 406 | 59 | 23 | 15 | 398 | 10 | SC beta-cypermethrin or SC deltamethrin  /malathion | Simple |
|  | 27 | nov-10 | 394 | 69 | 7 | 4 | 375 | 3 | SC beta-cypermethrin or SC deltamethrin | Simple |
|  | 39 | nov-11 | 387 | 75 | 24 | 24 | 338 | 12 | SC beta-cypermethrin or SC deltamethrin | Double |
|  | 51 | nov-12 | 380 | 78 | 31 | 26 | 359 | 11 | SC beta-cypermethrin or SC deltamethrin | Simple |
|  | 75 | nov-14 | 382 | 78 | 0 | 36 | 333 | 0 | None | - |
|  | 92 | apr-16 | 409 | 78 | 44 | 42 | 327 | 0 | None | - |
| 3 ^d^ | 0 | oct-08 | 409 | 17 | 0 | 0 | 408 | 413^e^ | SC beta-cypermethrin or SC deltamethrin | Simple |
|  | 10 | aug-09 | 425 | 26 | 8 | 32 | 414 | 46 | SC beta-cypermethrin or SC deltamethrin | Simple |
|  | 18 | apr-10 | 416 | 41 | 23 | 29 | 409 | 2 | SC deltamethrin | Simple |
|  | 38 | dec-11 | 424 | 43 | 27 | 37 | 86 | 2 | SC deltamethrin | Double |
|  | 49 | nov-12 | 438 | 47 | 39 | 58 | 436 | 3 | SC deltamethrin | Simple |
|  | 78 | apr-15 | 449 | 50 | 81 | 93 | 442 | 0 | None | - |
|  | 90 | apr-16 | 467 | 64 | 22 | 55 | 422 | 1 | SC beta-cypermethrin | Simple (domi), double (peri) |
| 4 | 0 | nov-09^f^/may-10/ nov-10^g^ | 269 | 23 | 0 | 0 | 260 | 239 | SC beta-cypermethrin or SC deltamethrin | Simple |
|  | 12 | nov-10^f,h^ | 173 | 11 | 2 | 9 | 158 | 3 | SC beta-cypermethrin or SC deltamethrin | Simple |
|  | 24 | nov-12^g,h^ | 21 | 14 | 0 | 1 | 17 | 0 | None | - |
|  | 37 | may-13/dec-13^g,h^ | 88 | 35 | 7 | 13 | 58 | 9 | SC deltamethrin | Simple |
|  | 42 | nov-12, may-13^f,h,i^ | 169 | 22 | 5 | 11 | 148 | 11 | SC deltamethrin | Simple |
|  | 60 | may-15^g,h^ | 81 | 42 | 2 | 2 | 54 | 2 | SC deltamethrin | Simple |
|  | 66 | may-15^f,h^ | 178 | 31 | 2 | 19 | 144 | 8 | SC deltamethrin | Simple |
|  | 71 | apr-16^g,h^ | 79 | 37 | 12 | 4 | 68 | 1 | SC beta-cypermethrin | Simple (domi), double (peri) |
|  | 77 | apr-16^f,h^ | 171 | 36 | 16 | 13 | 160 | 4 | SC beta-cypermethrin | Simple (domi), double (peri) |

^a^ A simple dose of suspension concentrate beta-cypermethrin is 50 mg/m^2^; for SC deltamethrin, 25 mg/m^2^.

^b^ Only new houses not sprayed at baseline.

^c^ A systematic sample.

^d^ A systematic sample at 59 MPI including 23 houses inspected, none sprayed, excluded from Fig 2–5 and 9.

^e^ Includes 26 houses sprayed by local healthcare agents in July 2008 and not re-sprayed five months later during the community-wide insecticide campaign.

^f^ Includes CY, CM and LM.

^g^ Includes TCZ, EP, H2 and CQ; only CQ and H2 surveyed at 24 MPI.

^h^ Data for successive surveys conducted at 12–24, 37–43, 60–66 and 71–77 MPI were pooled for a full coverage of area 4 villages at the midpoint of each period in Fig 2–3: 18, 40, 63 and 74 MPI).

^i^ Infestation data for 11 houses (4 sprayed with SC deltamethrin) conducted at 43 MPI (dec-2013) for selective (confirmatory) purposes were pooled with those recorded at 42 MPI and excluded from analyses.
